# Predictors of suicide attempt within 30 days of first medically documented major depression diagnosis in U.S. army soldiers with no prior suicidal ideation

**DOI:** 10.1186/s12888-023-04872-z

**Published:** 2023-06-02

**Authors:** Holly B. Herberman Mash, Robert J. Ursano, Ronald C. Kessler, James A. Naifeh, Carol S. Fullerton, Pablo A. Aliaga, Hieu M. Dinh, Nancy A. Sampson, Tzu-Cheg Kao, Murray B. Stein

**Affiliations:** 1 Center for the Study of Traumatic Stress, Department of Psychiatry, Uniformed Services University of the Health Sciences, 4301 Jones Bridge Road, Bethesda, MD 20814 USA; 2 Henry M. Jackson Foundation for the Advancement of Military Medicine, Inc., 6720A Rockledge Drive, Bethesda, MD 20817 USA; 3 Department of Health Care Policy, Harvard Medical School, 180 Longwood Avenue, 02115 Boston, MA USA; 4 Department of Preventive Medicine and Biostatistics, Uniformed Services University of the Health Sciences, 4301 Jones Bridge Road, Bethesda, MD 20814 USA; 5 Departments of Psychiatry and School of Public Health, University of California San Diego, 9500 Gilman Drive, La Jolla, 92093-0855 CA USA; 6 VA San Diego Healthcare System, 3350 La Jolla Village Drive, 92161 San Diego, CA USA; 7 Department of Psychiatry, Uniformed Services University of the Health Sciences, 4301 Jones Bridge Road, Bethesda, MD 20814 USA

**Keywords:** Suicide attempt, Suicidal behavior, Major depression diagnosis, Comorbid disorders, Military

## Abstract

**Background:**

Understanding mental health predictors of imminent suicide attempt (SA; within 30 days) among soldiers with depression and no prior suicide ideation (SI) can inform prevention and treatment. The current study aimed to identify sociodemographic and service-related characteristics and mental disorder predictors associated with imminent SA among U.S. Army soldiers following first documented major depression diagnosis (MDD) with no history of SI.

**Methods:**

In this case-control study using Army Study to Assess Risk and Resilience in Servicemembers (STARRS) administrative data, we identified 101,046 active-duty Regular Army enlisted soldiers (2010–2016) with medically-documented MDD and no prior SI (MDD/No-SI). We examined risk factors for SA within 30 days of first MDD/No-SI using logistic regression analyses, including socio-demographic/service-related characteristics and psychiatric diagnoses.

**Results:**

The 101,046 soldiers with documented MDD/No-SI were primarily male (78.0%), < 29 years old (63.9%), White (58.1%), high school-educated (74.5%), currently married (62.0%) and < 21 when first entering the Army (56.9%). Among soldiers with MDD/No-SI, 2,600 (2.6%) subsequently attempted suicide, 16.2% (n = 421) within 30 days (rate: 416.6/100,000). Our final multivariable model identified: Soldiers with less than high school education (χ^2^_3_ = 11.21, OR = 1.5[95%CI = 1.2–1.9]); combat medics (χ^2^_2_ = 8.95, OR = 1.5[95%CI = 1.1–2.2]); bipolar disorder (OR = 3.1[95%CI = 1.5–6.3]), traumatic stress (i.e., acute reaction to stress/not PTSD; OR = 2.6[95%CI = 1.4–4.8]), and “other” diagnosis (e.g., unspecified mental disorder: OR = 5.5[95%CI = 3.8-8.0]) diagnosed same day as MDD; and those with alcohol use disorder (OR = 1.4[95%CI = 1.0-1.8]) and somatoform/dissociative disorders (OR = 1.7[95%CI = 1.0-2.8]) diagnosed before MDD were more likely to attempt suicide within 30 days. Currently married soldiers (χ^2^_2_ = 6.68, OR = 0.7[95%CI = 0.6–0.9]), those in service 10 + years (χ^2^_3_ = 10.06, OR = 0.4[95%CI = 0.2–0.7]), and a sleep disorder diagnosed same day as MDD (OR = 0.3[95%CI = 0.1–0.9]) were less likely.

**Conclusions:**

SA risk within 30 days following first MDD is more likely among soldiers with less education, combat medics, and bipolar disorder, traumatic stress, and “other” disorder the same day as MDD, and alcohol use disorder and somatoform/dissociative disorders before MDD. These factors identify imminent SA risk and can be indicators for early intervention.

**Supplementary Information:**

The online version contains supplementary material available at 10.1186/s12888-023-04872-z.

## Background

The U.S. Army suicide rate increased substantially during the Iraq and Afghanistan wars, surpassing the age- and sex-adjusted civilian rate in 2008. Rates of suicidal behavior have since remained elevated [1–4]. Identification of factors predicting suicide attempt (SA) can improve clinical care for at-risk soldiers, particularly those not reporting suicidal ideation (SI) and therefore not identified at imminent risk. Much epidemiologic research examining SA risk uses survey data [5–8]. However, it is specifically important to consider SA risk in those with medically-documented psychiatric diagnoses [9], because they have been detected in the health care system. This information is critical for clinicians who, based on knowledge of patients’ current and past mental health, can identify at-risk patients who may benefit from early and rapid intervention.

Major depressive disorder (MDD) has consistently been associated with suicidal behavior [7, 10, 11]. Health care records of active-duty soldiers are generally comprehensive and capture all visits, offering unique opportunities to relate MDD to imminent (i.e., within 30 days) SA risk [12]. Predictors of imminent SA are particularly important because most individuals diagnosed with MDD do not attempt suicide [10, 13], and at-risk individuals may be difficult to identify, especially when SI is not detected.

Previous research using Army and Department of Defense (DoD) administrative data found that SAs are associated with socio-demographic characteristics [14], Army career characteristics, and psychiatric diagnosis [8, 15]. However, it is not known whether these factors distinguish soldiers with documented MDD who make subsequent SAs. Army studies using survey and administrative data suggest that transition from ideation to attempt is often rapid [16, 17], with most SAs occurring within one year [8, 18]. However, examination of transition from initial MDD to SA in soldiers without prior documented SI has not been examined using health care information.

Individuals with MDD often have comorbid psychiatric disorders [19]. Identifying co-occurring diagnoses that increase SA risk can distinguish who will rapidly transition to SA after initial MDD diagnosis. MDD and comorbid anxiety, posttraumatic stress (PTSD), substance, and personality disorders have been associated with suicide risk [20, 21]. However, most studies examining SA risk among depressed individuals focus on lifetime diagnoses or SA predictors over years, do not identify SA predictors at time of first depression diagnosis, and do not examine risk factors for rapid transition to SA particularly among those without SI.

Using administrative data from the Army Study to Assess Risk and Resilience in Servicemembers (STARRS) [22], this study examines imminent SA risk in soldiers with MDD and no documented SI (same-day or at any time during service before MDD diagnosis; MDD/No-SI). We identify the proportion of soldiers with depression who subsequently attempt suicide, the period of highest risk following first MDD diagnosis, and then examine socio-demographic/service-related characteristics and psychiatric diagnoses predicting SA within 30 days of MDD diagnosis.

## Methods

### Sample

The STARRS Historical Administrative Data Study (HADS) integrates 38 Army and DoD administrative data systems capturing medically-documented suicidal events and medical, legal, and personnel information during military service. The HADS includes individual-level person-month records for all Regular Army soldiers between January 1, 2010-December 31, 2016 [23]. Analysis of de-identified data was approved by Institutional Review Boards of STARRS-collaborating institutions and all methods were carried out in accordance with relevant guidelines and regulations.

The HADS contains administrative records for 918,281 Regular enlisted Army soldiers during the study period (excluding activated Army National Guard/Reserve). The analytic sample consisted of 101,046 Regular enlisted soldiers who were seen by a health care provider in a medical setting and received their first medically-documented diagnosis of MDD/no-SI (diagnosed before or same-day of depression diagnosis).

### Measures (Supplement includes full description)

**SI and SA** Soldiers with MDD/No-SI and those with SA within 30 days of first MDD were identified. Soldiers attempting suicide within 30 days of MDD diagnosis were ‘cases’ and those who did not were ‘controls.’ Incidence of subsequent SA within the study period (i.e., maximum seven years following MDD) was also identified. Classification used administrative records from: the DoDSER [24], a DoD-wide surveillance mechanism, and ICD-9-CM V62.84 and ICD-10-CM R45.851 codes (SI), ICD-9-CM E950-E958 (self-inflicted poisoning/injury with suicidal intent) and ICD-10-CM X71-X83 (intentional self-harm), T36-T65, T71 (where 5th and 6th characters indicate intentional self-harm), and T14.91 (SA) codes [25] from health care information from military and civilian treatment facilities, combat operations, and aeromedical evacuations (Table S1, online: www.starrs-ls.org/#/list/publications). SI was excluded based on either having an ICD-9/ICD-10 code or a DoDSER record indicating SI.

#### Socio-demographic and service-related characteristics

Personnel records were used to construct socio-demographic (gender, current age, race/ethnicity, education, marital status) and service-related variables (age at Army entry, time in service, deployment status, demotion, delayed promotion, and military occupation; Table S2).

#### Psychiatric diagnosis

Administrative medical records identified 26 documented psychiatric diagnostic categories defined by aggregated ICD-9-CM and ICD-10-CM codes and ICD-9-CM V and ICD-10-CM Z stressors/adversities and marital problems codes (Table S3). Each diagnostic category was coded into two time periods: occurring same-day as first MDD diagnosis and occurring any time during service before first MDD diagnosis.

### Statistical analysis

Analyses were conducted using SAS version 9.4 [26]. Associations of all socio-demographic/service-related characteristics and psychiatric diagnoses with SA within 30 days of depression diagnosis were examined using univariable logistic regression. Multivariable logistic regression analyses were conducted for each psychiatric diagnosis, adjusting for socio-demographics/service-related characteristics. A final model was conducted including diagnoses significant in the separate multivariable analyses. This model-building approach was based on purposeful factor selection to identify the most parsimonious model including all relevant predictors [26]. The significance threshold for all analyses was *p* < .05, with selected variables for the final model identified by this criterion [27].

Logistic regression coefficients were exponentiated to obtain odds ratios (OR) and 95% confidence intervals (CI). To account for secular trends, logistic regression equations controlled for calendar month and year. Coefficients of other predictors can consequently be interpreted as averaged within-month associations based on the assumption that other predictors’ effects do not vary over time. Diagnostic performance of the final model using risk prediction was evaluated calculating positive predictive value (PPV) among the 10% of participants at highest predicted risk. Population-attributable risk proportion (PARP) [28] was calculated to identify the proportion of observed SAs that would not occur if effects attributable to specific mental disorders were reduced to reference level (i.e., from high risk level (top 10%) based on PPV to medium risk level (middle 30%)), assuming that model coefficients represent causal effects of the predictors.

## Results

Soldiers with documented MDD/No-SI (*n* = 101,046) were primarily male (78.0%), < 29 years (63.9%), White (58.1%), high school-educated (74.5%), currently married (62.0%), and < 21 when first entering Army (56.9%) (Table 1). Approximately one-third (31.1%) had 5–10 years of service, 60.5% had previously deployed, and 20.5% were assigned to combat arms. The five most common psychiatric diagnostic categories among soldiers with MDD/No-SI were: stressors/adversities and marital problems (46.6% before day of depression diagnosis); tobacco use disorder (41.7% before depression); anxiety disorder (41.2% before depression); adjustment disorder (25.2% before depression); and dysthymic disorder/neurasthenia/depression NOS (24.6% before depression)) (Table S4).

**Table 1:** Association of socio-demographic and service-related characteristics of active-duty Regular U.S. Army enlisted soldiers with documented suicide attempt within 30 days following initial major depression diagnosis and no prior suicidal ideation

		Univariable		Soldiers with Major Depression and No Prior Suicidal Ideation				Total population (n = 101,046)
				Attempted suicide within 30 days^a^ (n = 421)		Did not attempt suicide within 30 days^b^ (n = 100,625)		
	χ²	OR^c^	(95% CI^c^)	n	%	n	%	%
**Socio-demographic Characteristics**								
Gender								
Male		1.0	–	313	74.3	78,467	78.0	78.0
Female		1.2	(1.0–1.5)	108	25.7	22,158	22.0	22.0
	3.26							
Current Age								
< 21		6.1*	(4.0–9.3)	117	27.8	10,756	10.7	10.8
21–24		3.3*	(2.2–4.9)	161	38.2	27,759	27.6	27.6
25–29		2.1*	(1.4–3.3)	97	23.0	25,623	25.5	25.5
30–34		1.0	–	27	6.4	15,318	15.2	15.2
35–39		0.5	(0.3–1.1)	10	2.4	10,687	10.6	10.6
40+		0.5	(0.2–1.0)	9	2.1	10,482	10.4	10.4
	163.91*							
Race/Ethnicity								
White		1.0	–	270	64.1	58,389	58.0	58.1
Black		0.8*	(0.6–1.0)	87	20.7	24,236	24.1	24.1
Hispanic		0.9	(0.7–1.2)	50	11.9	12,106	12.0	12.0
Asian		0.4*	(0.2–0.8)	9	2.1	4,756	4.7	4.7
Other		0.9	(0.4–2.3)	5	1.2	1,138	1.1	1.1
	10.12*							
Education								
< High school^d^		1.6*	(1.3–2.0)	93	22.1	14,188	14.1	14.1
High school		1.0	–	310	73.6	74,951	74.5	74.5
Some college		0.3*	(0.2–0.7)	7	1.7	5,328	5.3	5.3
≥College		0.4*	(0.2–0.8)	11	2.6	6,158	6.1	6.1
	34.58*							
Marital Status								
Never married		1.0	–	231	54.9	32,629	32.4	32.5
Currently married		0.4*	(0.3–0.5)	177	42.0	62,428	62.0	62.0
Previously married		0.3*	(0.2–0.6)	13	3.1	5,568	5.5	5.5
	89.34*							
**Service-related Characteristics**								
Age at Army entry								
< 21		1.2	(0.9–1.5)	273	64.8	57,183	56.8	56.9
21–24		1.0	–	106	25.2	26,125	30.0	26.0
25+		0.6*	(0.4–0.9)	42	10.0	17,317	17.2	17.2
	17.00*							
Time in Service								
1–2 years		2.8*	(2.2–3.6)	187	44.4	20,433	20.3	20.4
3–4 years		1.4*	(1.1–1.9)	117	27.8	25,083	24.9	24.9
5–10 years		1.0	–	101	24.0	31,372	31.2	31.1
> 10 years		0.2*	(0.1–0.4)	16	3.8	23,737	23.6	23.5
	149.43*							
Deployment Status								
Never		1.0	–	219	52.0	33,412	33.2	33.3
Current		0.9	(0.6–1.2)	36	8.6	6,204	6.2	6.2
Previous		0.4*	(0.3–0.5)	166	39.4	61,009	60.6	60.5
	75.42*							
Demotion								
Past year		1.5*	(1.1–2.2)	33	7.8	5,225	5.2	5.2
Before past year		0.9	(0.6–1.3)	37	8.8	9,859	9.8	9.8
Never demoted		1.0	–	351	83.4	85,541	85.0	85.0
	5.99*							
Delayed Promotion								
On Schedule		1.0	–	160	38.0	16,404	16.3	16.4
Late: </= 2 months		0.5	(0.2–1.4)	4	0.9	820	0.8	0.8
Late: > 2 months		0.6*	(0.4–0.9)	37	8.8	5,970	5.9	5.9
Not relevant due to rank^e^		0.3*	(0.2–0.4)	220	52.3	77,431	77.0	76.8
	141.23*							
Military Occupational Specialty (MOS)								
Combat Arms^f^		1.5*	(1.2–1.8)	112	26.6	20,561	20.4	20.5
Combat Medics		1.5*	(1.1–2.1)	35	8.3	6,288	6.2	6.3
Other MOS		1.0	–	274	65.1	73,776	73.3	73.3
	14.17*							

^a^Soldiers with first-time documented major depression and no prior suicidal ideation who subsequently attempted suicide within the next 30 days of recorded major depression

^b^Soldiers with first-time documented major depression and no prior suicidal ideation who did not subsequently attempt suicide within the next 30 days

^c^OR = Odds ratio; CI = Confidence interval

^d^< High School includes: General Educational Development credential (GED), home study diploma, occupational program certificate, correspondence school diploma, high school certificate of attendance, adult education diploma, and other non-traditional high school credentials

^e^Soldiers above the rank of E4 are not promoted on a set schedule

^f^Combat Arms includes Combat Arms and Special Forces soldiers

*p < .05

**Fig. 1:**
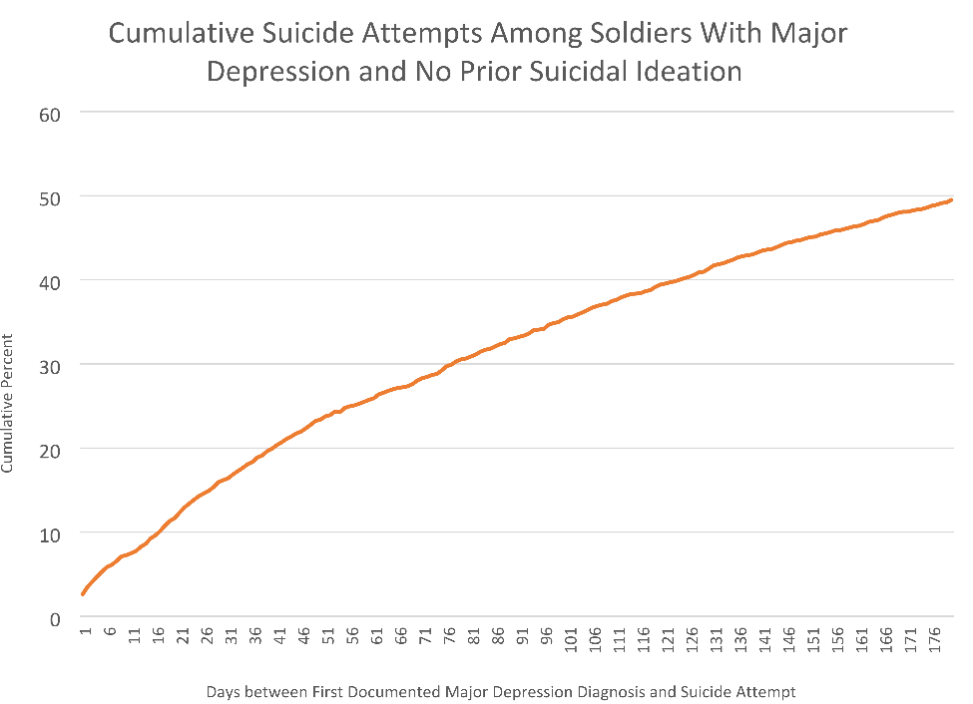
Cumulative percent of suicide attempts across days since first documented major depression diagnosis

**Fig. 2:**
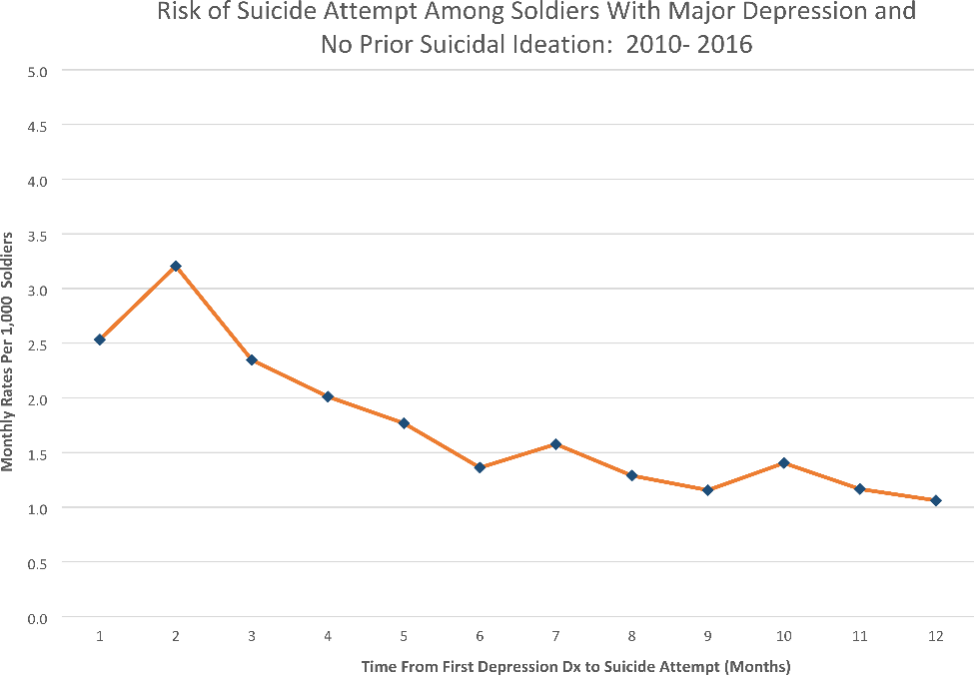
Risk of suicide attempt among Regular Army-enlisted soldiers following first documented major depression diagnosis

Among the soldiers with first documented MDD/No-SI, 2,600 (2.6%) subsequently attempted suicide (i.e., maximum seven years following MDD). Nearly 50% (*n* = 1287) of SAs occurred within 180 days of MDD diagnosis (Fig. 1), with 16.2% (*n* = 421) occurring within the first 30 days (or 1/3 of those attempting suicide within 180 days). Figure 2 shows the hazard function indicating highest SA risk in the second month after MDD (rate:3.2/1,000 soldiers) with incrementally decreasing risk over time. This rate is > 10 times higher than the annual SA rate previously reported [29]. Examination by day showed the first day after MDD/No-SI diagnosis with highest daily risk (67.3/100,000 soldiers).

### Socio-demographic and service-related risk factors

In univariable analyses, younger soldiers were more likely to attempt suicide. Soldiers < 21 were over six times more likely than those 30–34 to attempt suicide (χ^2^_5_ = 163.91, OR = 6.1[95%CI = 4.0-9.3]) (Table 1). SA was more likely among soldiers with less than high school education (χ^2^_3_ = 34.58, OR = 1.6[95%CI = 1.3-2.0]), and less likely if soldiers were Black and Asian (χ^2^_4_ = 10.12; OR = 0.8[95%CI = 0.6-1.0] and OR = 0.4[95%CI = 0.2–0.8], respectively). Gender was not associated with SA risk.

Soldiers who were 25 + years when entering the Army (χ^2^_2_ = 17.00, OR = 0.6[95%CI = 0.4–0.9]), were previously deployed (χ^2^_2_ = 75.42, OR = 0.4[95%CI = 0.3–0.5]), and were promoted 2 + months late (χ^2^_3_ = 141.23, OR = 0.6[95%CI = 0.4–0.9]) were less likely to attempt suicide. Soldiers with fewer years of service (χ^2^_3_ = 149.43, 1–2 years: OR = 2.8[95%CI = 2.2–3.6]; 3–4 years: OR = 1.4[95%CI = 1.1–1.9]), were demoted in past year (χ^2^_2_ = 5.99, OR = 1.5[95%CI = 1.1–2.2]), and were combat arms or combat medics (χ^2^_2_ = 14.17; OR = 1.5[95%CI = 1.2–1.8] and OR = 1.5[95%CI = 1.1–2.1], respectively) were more likely to attempt suicide.

### Psychiatric diagnosis

Univariable analyses indicated that soldiers diagnosed with bipolar disorder same-day as first MDD diagnosis were > 7 times more likely to attempt suicide within 30 days (OR = 7.1[95%CI = 3.6–13.8]) (Table 2; Table S4). A same-day diagnosis from “other” category was also associated with increased SA odds (OR = 10.3[95%CI = 7.3–14.5]). The most common diagnosis in “other” category was ICD-9-CM code 300.9 (unspecified nonpsychotic mental disorder), diagnosed in 77% (*n* = 746/969) of soldiers with “other” diagnosis and 89.2% (*n* = 33/37) of suicide attempters with “other” diagnosis. Additional disorders diagnosed same-day as depression and associated with SA risk were personality disorder (OR = 4.3[95%CI = 2.2–8.3]), non-affective psychosis (OR = 3.5[95%CI = 1.8–6.7]), traumatic stress (i.e., acute reaction to stress/not PTSD; OR = 3.3[95%CI = 1.8–3.3]), drug-induced mental disorders (OR = 2.6[95%CI = 1.1–6.2]), dysthymic disorder/neurasthenia/depression NOS (OR = 2.4[95%CI = 1.7–3.4]), adjustment disorder (OR = 2.1[95%CI = 1.4–3.1]), tobacco use disorder (OR = 1.7[95%CI = 1.2–2.4]), stressors/adversities and marital problems (OR = 1.4[95%CI = 1.0-1.8]), and anxiety disorder (OR = 1.3[95%CI = 1.0-1.7]). Alcohol use disorder, diagnosed both same-day (OR = 1.9[95%CI = 1.3–2.6]) and before depression (OR = 1.4[95%CI = 1.1–1.8]), was also associated with SA risk.

**Table 2:** Association of psychiatric diagnoses of active-duty Regular U.S. Army enlisted soldiers with documented suicide attempt within 30 days following initial major depression diagnosis

		Univariable			Multivariable^c^		Soldiers with Major Depression Diagnosis with No SI				Total population (*n* = 101,046)
							Attempt suicide within 30 days^a^ (*n* = 421)		Did not attempt suicide within 30 days^b^ (*n* = 100,625)		
	χ²	OR^d^	(95% CI^d^)	χ²	OR	(95% CI)	*n*	%	*n*	%	%
**Psychiatric Diagnosis**											
Adjustment Disorder (Day of depression diagnosis)											
Yes		2.1*	(1.4–3.1)		1.6*	(1.1–2.4)	26	6.2	3,119	3.1	3.1
No		1.0	–		1.0	–	395	93.8	97,506	96.9	96.9
	12.57*			5.39*							
Alcohol Use Disorder (Day of depression diagnosis)											
Yes		1.9*	(1.3–2.6)		1.7*	(1.2–2.4)	40	9.5	5,380	5.3	5.4
No		1.0	–		1.0	–	381	90.5	95,245	94.7	94.6
	13.94*			10.36*							
Alcohol Use Disorder (Prior to depression diagnosis)											
Yes		1.4*	(1.1–1.8)		1.5*	(1.2–2.0)	74	17.6	13,461	13.4	13.4
No		1.0	–		1.0	–	347	82.4	87,164	86.6	86.6
	6.41*			9.85*							
Anxiety Disorders (Day of depression diagnosis)											
Yes		1.3*	(1.0–1.7)		1.4*	(1.1–1.7)	93	22.1	17,992	17.9	17.9
No		1.0	–		1.0	–	328	77.9	82,633	82.1	82.1
	5.21*			6.50*							
Anxiety Disorders (Prior to depression diagnosis)											
Yes		0.7*	(0.6–0.9)		1.0	(0.8–1.2)	142	33.7	41,470	41.2	41.2
No		1.0	–		1.0	–	279	66.3	59,155	58.8	58.8
	9.24*			0.00							
Bipolar Disorder (Day of depression diagnosis)											
Yes		7.1*	(3.6–13.8)		5.3*	(2.7–10.4)	9	2.1	307	0.3	0.3
No		1.0	–		1.0	–	412	97.9	100,318	99.7	99.7
	32.71*			23.43*							
Dysthymic Disorder/Neurasthesia/ Depression NOS (Day of depression diagnosis)											
Yes		2.4*	(1.7–3.4)		1.8*	(1.3–2.5)	38	9.0	3,950	3.9	3.9
No		1.0	–		1.0	–	383	91.0	96,675	96.1	96.1
	26.87*			11.90*							
Stressors/Adversities & Marital Problems (Day of depression diagnosis)											
Yes		1.4*	(1.0–1.8)		1.3*	(1.0–1.8)	60	14.3	10,831	10.8	10.8
No		1.0	–		1.0	–	361	85.7	89,794	89.2	89.2
	5.32*			4.36*							
Stressors/Adversities & Marital Problems (Prior to depression diagnosis)											
Yes		0.7*	(0.6–0.8)		1.0	(0.8–1.2)	157	37.3	46,943	46.7	46.6
No		1.0	–		1.0	–	264	62.7	53,682	53.3	53.4
	14.08*			0.01							
Non-Affective Psychosis (Day of depression diagnosis)											
Yes		3.5*	(1.8–6.7)		2.6*	(1.3–5.0)	9	0.2	633	0.6	0.6
No		1.0	–		1.0	–	412	97.9	99,992	99.4	99.4
	13.39*			7.60*							
Other Diagnosis (Day of depression diagnosis)^e^											
Yes		10.3*	(7.3–14.5)		7.0*	(4.9–9.9)	37^e^	8.8	932	0.9	1.0
No		1.0	–		1.0	–	384	91.2	99,693	99.1	99.0
	176.84*			118.47*							
Personality Disorder (Day of depression diagnosis)											
Yes		4.3*	(2.2–8.3)		3.2*	(1.7–6.3)	9	0.2	510	0.5	0.5
No		1.0	–		1.0	–	412	97.9	100,115	99.5	99.5
	18.28*			11.78*							
Sexual Disorder (Prior to depression diagnosis)											
Yes		0.2*	(0.1–0.5)		0.6	(0.2–1.5)	4	1.0	4,671	4.6	4.6
No		1.0	–		1.0	–	417	99.0	95,954	95.4	95.4
	10.38*			0.13							
Sleep Disorder (Day of depression depression)											
Yes		0.3*	(0.1–0.8)		0.4*	(0.1–1.0)	4	1.0	3,028	3.0	3.0
No		1.0	–		1.0	–	417	99.0	97,597	97.0	97.0
	5.49*			3.95*							
Sleep Disorder (Prior to depression diagnosis)											
Yes		0.7*	(0.6–1.0)		1.2	(0.9–1.6)	52	12.4	16,110	16.0	16.0
No		1.0	–		1.0	–	369	87.6	84,515	84.0	84.0
	4.05*			0.99							
Somatoform/Dissociative Disorder (Prior to depression diagnosis)											
Yes		1.1	(0.7–1.8)		1.7*	(1.0–2.8)	17	4.0	3,768	3.7	3.7
No		1.0	–		1.0	–	404	96.0	96,857	96.3	96.3
	0.10			4.60*							
Tobacco Use Disorder (Day of depression diagnosis)											
Yes		1.7*	(1.2–2.4)		1.7*	(1.2–2.3)	41	9.7	5,949	5.9	5.9
No		1.0	–		1.0	–	380	90.3	94,676	94.1	94.1
	10.82*			9.18*							
Traumatic Stress (Day of depression diagnosis)											
Yes		3.3*	(1.8–6.0)		2.9*	(1.6–5.2)	11	2.6	817	0.8	0.8
No		1.0	–		1.0	–	410	97.4	99,808	99.2	99.2
	14.76*			11.46*							
PTSD (Prior to depression diagnosis)											
Yes		0.7*	(0.5–0.9)		1.2	(0.9–1.7)	43	10.2	14,602	14.5	14.5
No		1.0	–		1.0	–	378	89.8	86,023	85.5	85.5
	6.05*			1.52							
Drug Induced Mental Disorder (Day of depression diagnosis)											
Yes		2.6*	(1.1–6.2)		2.0	(0.8–4.9)	5	1.2	467	0.5	0.5
No		1.0	–		1.0	–	416	98.8	100,158	99.5	99.5
	4.35*			2.30							
Drug Induced Mental Disorder (Prior to depression diagnosis)											
Yes		1.5	(0.9–2.5)		1.7*	(1.0–2.9)	16	3.8	2,512	2.5	2.5
No		1.0	–		1.0	–	405	96.2	98,113	97.5	97.5
	2.83			4.39*							
Non-Dependent Drug Abuse (Day of depression diagnosis)											
Yes		2.3*	(1.3–4.2)		1.6	(0.9–3.0)	11	2.6	1,152	1.1	1.2
No		1.0	–		1.0	–	410	97.4	99,473	98.9	98.8
	7.41*			2.50							

^a^Cases: Soldiers with first-time documented major depression with no prior suicidal ideation who subsequently attempted suicide within the next 30 days of recorded depression diagnosis

^b^Controls: Soldiers with first-time documented major depression with no prior suicidal ideation who did not subsequently attempt suicide within the next 30 days

^c^Each variable was examined in a separate multivariable model that adjusted for socio-demographics (gender, current age, race, education, and marital status) and service-related characteristics (age at entry into Army service, time in service, deployment status, delayed promotion, demotion, and military occupation)

^d^OR = Odds ratio; CI = Confidence interval

^e^Among soldiers who were diagnosed with a disorder classified as “Other,” 33/37 (89.2%) were identified by the ICD-9-CM code 300.9 (Unspecified nonpsychotic mental disorder)

*p < .05

Soldiers were less likely to attempt suicide if diagnosed with the following disorders before their depression diagnosis: anxiety disorder (OR = 0.7[95%CI = 0.6–0.9]); PTSD (OR = 0.7[95%CI = 0.5–0.9]); stressors/adversities and marital problems (OR = 0.7[95%CI = 0.6–0.8]); sexual disorders (OR = 0.2[95%CI = 0.1–0.5]); and sleep disorders (OR = 0.7[95%CI = 0.6-1.0]. Soldiers were also less likely to attempt suicide if they were diagnosed with sleep disorder on the same day (OR = 0.3[95%CI = 0.1–0.8]).

### Multivariable analyses

A multivariable model with socio-demographic/service-related characteristics indicated SAs within 30 days were more likely if soldiers with MDD/No-SI were < 21 (χ^2^_5_ = 7.8, OR = 2.4[95%CI = 1.2–4.9]), completed less than high school (χ^2^_3_ = 13.2, OR = 1.5[95%CI = 1.2–1.9]), had been demoted before the past year (χ^2^_2_ = 5.7, OR = 1.5[95%CI = 1.0-2.1]), and were combat arms or combat medics (χ^2^_2_ = 9.6; OR = 1.4[95%CI = 1.1–1.7] and OR = 1.5[95%CI = 1.0-2.1], respectively) (Table S5). SAs were less likely if soldiers were currently married (χ^2^_2_ = 8.1, OR = 0.7[95%CI = 0.6–0.9]) and had 10 + years of military service (χ^2^_3_ = 10.5, OR = 0.4[95%CI = 0.2–0.7]).

A series of separate multivariable models examining each specific psychiatric diagnosis, adjusting for socio-demographic/service-related variables, indicated that soldiers diagnosed with bipolar disorder same-day as depression were > 5 times more likely to attempt suicide within 30 days (OR = 5.3[95%CI = 2.7–10.4]). Further, those diagnosed with a mental disorder identified as “other” the same day were seven times as likely to attempt suicide (OR = 7.0[95%CI = 4.9–9.9]) (Table 2 and S6). Additional diagnoses given same-day as depression and associated with SA included: personality disorders (OR = 3.2[95%CI = 1.7–6.3]), tobacco use disorder (OR = 1.7[95%CI = 1.2–2.3]), non-affective psychosis (OR = 2.6[95%CI = 1.3-5.0]), dysthymic disorder/neurasthenia/depression NOS (OR = 1.8[95%CI = 1.3–2.5]), adjustment disorder (OR = 1.6[95%CI = 1.1–2.4]), alcohol use disorder (OR = 1.7[95%CI = 1.2–2.4]), anxiety disorders (OR = 1.4[95%CI = 1.1–1.7]), and stressors/adversities and marital problems (OR = 1.3[95%CI = 1.0-1.8]). Soldiers with sleep disorder diagnosed same-day as depression were less likely to attempt suicide (OR = 0.4[95%CI = 0.1-1.0]).

Soldiers with a documented somatoform/dissociative disorder (OR = 1.7[95%CI = 1.0-2.8]), drug-induced mental disorders (OR = 1.7[95%CI = 1.0-2.9]), and alcohol use disorder (OR = 1.5[95%CI = 1.2-2.0]) before depression were also more likely to attempt suicide.

The final model included all socio-demographic/service-related characteristics and the 15 psychiatric disorders diagnosed same-day and before depression diagnosis that were significant in the separate multivariable models (Table 3). In this model, having less than high school education (χ^2^_3_ = 11.2; OR = 1.5[95%CI = 1.2–1.9]) and being a combat medic (χ^2^_2_ = 9.0; OR = 1.5[95%CI = 1.1–2.2]) were associated with greater SA risk, and being currently married (χ^2^_2_ = 6.7; OR = 0.7[95%CI = 0.6–0.9]) and in service 10 + years (χ^2^_3_ = 10.1; OR = 0.4[95%CI = 0.2–0.7]) were associated with lower risk. Same-day diagnosis of bipolar disorder (OR = 3.1[95%CI = 1.5–6.3]), traumatic stress (OR = 2.6[95%CI = 1.4–4.8]), and “other” diagnosis (OR = 5.5[95%CI = 3.8-8.0]), and prior alcohol use disorder (OR = 1.4[95%CI = 1.0-1.8]) and somatoform/dissociative disorders (OR = 1.7[95%CI = 1.0-2.8]) diagnoses continued to be associated with increased SA risk. Same-day sleep disorder diagnosis (OR = 0.3[95%CI = 0.1–0.9]) was associated with lower risk. Ten soldiers with MDD/No-SI died by suicide within 30 days. When we added soldier deaths to our cases, findings in our final model were unchanged.

**Table 3:** Multivariable associations of psychiatric diagnosis in active-duty Regular U.S. Army enlisted soldiers with documented suicide attempt within 30 days following initial major depression diagnosis and no prior suicidal ideation^a^

	χ²	OR	(95% CI)
**Socio-demographic characteristics**			
Gender			
Male		1.0	–
Female		1.2	(0.9–1.5)
	1.51		
Current Age			
< 21		2.4	(1.2–4.9)
21–24		1.7	(1.0–3.2)
25–29		1.4	(0.9–2.4)
30–34		1.0	–
35–39		0.8	(0.4–1.7)
40+		0.9	(0.4–2.1)
	7.81		
Race/ethnicity			
White		1.0	–
Black		1.0	(0.8–1.3)
Hispanic		1.1	(0.8–1.5)
Asian		0.6	(0.3–1.1)
Other		1.1	(0.4–2.6)
	3.30		
Education			
< High school^c^		1.5*	(1.2–1.9)
High school		1.0	–
Some college		0.7	(0.3–1.5)
≥ College		0.9	(0.5–1.8)
	11.21*		
Marital status			
Never married		1.0	–
Currently married		0.7*	(0.6–0.9)
Previously married		0.9	(0.5–1.6)
	6.68*		
**Service-related characteristics**			
Age at Army Entry			
< 21		0.9	(0.7–1.2)
21–24		1.0	–
25+		0.9	(0.6–1.5)
	0.34		
Time in Service			
1–2 years		1.5	(0.9–2.7)
3–4 years		1.1	(0.8–1.6)
5–10 years		1.0	–
> 10 years		0.4*	(0.2–0.7)
	10.06*		
Deployment Status			
Never		1.0	–
Currently		1.5	(1.0–2.2)
Previously		1.0	(0.8–1.4)
	4.67		
Demotion			
Past year		1.3	(0.8–2.0)
Before past year		1.4	(0.9–2.0)
Never demoted		1.0	–
	3.24		
Delayed Promotion			
On schedule		1.0	–
Late: </= 2 months		0.6	(0.2–1.6)
Late: > 2 months		0.7	(0.4–1.3)
Not relevant due to rank^d^		0.8	(0.5–1.3)
	2.02		
Military Occupational Specialty (MOS)			
Combat Arms^e^		1.3	(1.0–1.7)
Combat Medics		1.5*	(1.1–2.2)
Other MOS		1.0	–
	8.95*		
**Psychiatric diagnosis**			
Adjustment Disorder (Day of depression diagnosis)			
Yes		1.3	(0.9–1.9)
No		1.0	–
	1.41		
Alcohol Use Disorder (Day of depression diagnosis)			
Yes		1.2	(0.8–1.7)
No		1.0	–
	0.90		
Alcohol Use Disorder (Prior to depression diagnosis)			
Yes		1.4*	(1.0–1.8)
No		1.0	–
	3.95*		
Anxiety Disorders (Day of depression diagnosis)			
Yes		1.3	(1.0–1.6)
No		1.0	–
	3.43		
Bipolar Disorder (Day of depression diagnosis)			
Yes		3.1*	(1.5–6.3)
No		1.0	–
	9.71*		
Dysthymic Disorder / Neurasthenia / Depression NOS (Day of depression diagnosis)			
Yes		1.4	(1.0–2.0)
No		1.0	–
	3.18		
Stressors/Adversities & Marital Problems (Day of depression diagnosis)			
Yes		1.1	(0.8–1.5)
No		1.0	–
	0.45		
Non-Affective Psychosis (Day of depression diagnosis)			
Yes		1.4	(0.7–2.7)
No		1.0	–
	0.77		
Other Diagnosis (Day of depression diagnosis)^f^			
Yes		5.5*	(3.8–8.0)
No		1.0	–
	82.71*		
Personality Disorder (Day of depression diagnosis)			
Yes		1.7	(0.8–3.4)
No		1.0	–
	1.95		
Sleep Disorder (Day of depression diagnosis)			
Yes		0.3*	(0.1–0.9)
No		1.0	–
	4.40*		
Somatoform/Dissociative Disorders (Prior to depression diagnosis)			
Yes		1.7*	(1.0–2.8)
No		1.0	–
	4.36*		
Tobacco Use Disorder (Day of depression diagnosis)			
Yes		1.2	(0.9–1.7)
No		1.0	–
	1.25		
Traumatic Stress (Day of depression diagnosis)			
Yes		2.6*	(1.4–4.8)
No			–
	9.26*		
Drug Induced Mental Disorder (Prior to depression diagnosis)			
Yes		1.5	(0.9–2.5)
No		1.0	–
	2.15		

^a^All variables that were significant in separate multivariable models that adjusted for socio-demographics (gender, current age, race, education, and marital status) and service-related characteristics (age at entry into Army service, time in service, deployment status, delayed promotion, demotion, military occupation) were examined together in a final multivariable model

^b^OR = Odds ratio; CI = Confidence interval

^c^< High School includes: General Educational Development credential (GED), home study diploma, occupational program certificate, correspondence school diploma, high school certificate of attendance, adult education diploma, and other non-traditional high school credentials

^d^Soldiers above the rank of E4 are not promoted on a set schedule

^e^Combat Arms includes Combat Arms and Special Forces soldiers

^f^Among soldiers who were diagnosed with a disorder classified as “Other,” 33/37 (89.2%) were identified by the ICD-9-CM code 300.9 (Unspecified nonpsychotic mental disorder)

*p < .05

Using predicted probabilities from this model, the 10% of participants with highest predicted SA risk included 37.1% of participants with SA (sensitivity = 37.1%). The PARP based on our final model was 25.2%, suggesting SA might be reduced by as much as one-quarter if we could intervene with this group and reduce their risk from high to medium risk level.

## Discussion

Identifying individuals at imminent SA risk is a difficult and important clinical task when depression is diagnosed. This is especially challenging among individuals with MDD when SI is not detected during evaluation. The current study’s focus on factors distinguishing soldiers with MDD/No-SI who attempt suicide within 30 days identifies targets for clinicians providing care at the time of first depression diagnosis. This focus can also help us understand progression from depression diagnosis to SA by identifying lifetime and current mental disorder diagnoses specifically related to rapid transition from MDD to SA. This is particularly important in the military, given that suicide has been increasing, despite efforts to reduce its prevalence. The STARRS HADS provides a unique opportunity to examine comprehensive administrative medical records during soldiers’ time in service, which has not been similarly explored in civilian samples.

In this study, 2.6% of soldiers with MDD/No-SI subsequently attempted suicide, with 16.2% of attempts occurring within 30 days of first depression diagnosis. SA risk was highest early after depression diagnosis and decreased over time, highlighting the importance of risk assessment and identifying those at high SA risk at initial depression diagnosis.

Soldiers with less than high school education and combat medics were at increased risk of imminent SA. In contrast, currently married soldiers and those in service 10 + years were less likely to attempt. Similar demographic [4, 29] and service-related findings [15, 29, 30] have been identified. Combat medics are at elevated SA risk during first year of service [30], possibly associated with advanced training and performance demands. Combat medics were also identified at high SA risk within 30 days after SI diagnosis [16], further emphasizing need for attention to SA prevention among this group. Of importance, our sample differs from this previous study examining the 2004–2009 period, further informing risk in combat medics by identifying rapid transition to SA among medics with depression.

Contrary to research indicating overall population-level higher SA risk among female soldiers [15, 31], women with depression were not more likely to attempt suicide within 30 days. Given that depression is more common in women in both civilian [32] and military [33, 34] populations, further examination of psychiatric disorders associated with SA among Army women with various medically-documented diagnoses may clarify this relationship.

In this study, five psychiatric diagnoses were associated with attempt. Soldiers diagnosed with bipolar disorder, traumatic stress, and “other” disorder (identified among most soldiers in this diagnostic category as unspecified nonpsychotic mental disorder; Table S3) same-day as their first depression diagnosis, and those diagnosed with alcohol use disorder and somatoform/dissociative disorders before depression diagnosis, were more likely to attempt suicide within 30 days. The “other” diagnosis suggests that disorder-related symptoms, perhaps acute stress symptoms not specified nor meeting full criteria for a diagnosis, may be indicating the acute process and disorganized symptom pattern underlying rapid transitions to SA. These diagnostic categories should heighten clinical concern, and may reflect different levels of acute or chronic emotional dysregulation and presence of acute stressors.

Similar to our findings, other studies report that individuals with bipolar disorder who attempt suicide are more likely to experience a current depressive (or mixed state) episode [35]. Aggression and irritability predict SA among individuals with bipolar disorder [35] and often characterize agitated depression, reported as the highest-risk condition for suicidal behaviors [36]. Patients with bipolar disorder are most likely to attempt suicide during severe, pure, or mixed depressive episodes (78–89%) [36]. Patients with rapid cycling are at 54% higher SA risk [37]. Further consideration of related dimensional categories, including anger, irritability, and emotion dysregulation, may aid SA risk identification.

Traumatic stress (i.e., acute reaction to stress), but not PTSD, was associated with imminent SA risk. This acute stress response indicator suggests a recent life stressor and substantial symptom response. Our study did not examine specific life events/transitions (e.g., new assignments, transitions out of training, stressful duty assignments) occurring within 30 days, an important area for future study of rapid transition to SA.

Somatoform-related and/or dissociative disorders diagnoses before depression diagnosis were also associated with increased SA risk. Disorders characterized by somatic symptoms were associated with SAs in 13–67% of participants with somatic disorders [38]. Individuals seeking treatment for somatic symptoms often seek care from non-psychiatric providers [38, 39]. SA risk is noted among individuals with somatoform disorders who develop mood disorders [38]. Identifying the role of medication prescribed for physical symptoms and hopelessness associated with unresolved medical complaints may aid in understanding risk. Dissociative disorders, defined by another disorganizing set of symptoms seen with acute emotional dysregulation, have been associated with SA even when controlling for PTSD [40]. Dissociative symptoms and suicidal behaviors may reflect emotional dysregulation [41]. Dissociation is also related to problematic alcohol use in veterans [42], and associated in this study with increased imminent SA risk when diagnosed before depression.

Several limitations should be considered when interpreting study findings. First, this study used administrative records. Thus, identified cases are subject to classification/coding errors and limited to events receiving medical attention. Although the extent to which attempts are accurately captured in soldiers’ medical records, similar to civilian care settings, cannot be conclusively identified, a substantial number of at-risk soldiers were documented. Future analyses of STARRS survey data linked to respondents’ administrative records can clarify frequency of unidentified suicide attempts during service. Our study data focus on the 2010–2016 period; therefore, findings may not generalize to other time periods. Future research that replicates findings using different military cohorts and/or registries is recommended, and should include Army National Guard/Reserve soldiers and veterans. Research would also benefit from examination of the reasons for increased risk of suicide attempt among soldiers, including military-specific occupational risks and access to firearms, which are of public health importance.

Importantly, this study identified factors associated with acute SA risk among soldiers when first diagnosed with MDD/No-SI, highlighting the significance of rapid transition to SA and possible need for clinical intervention or intensive follow-up. Notably, given that only < 2% of studies examining suicide specifically focus on imminent risk factors [43], future research should consider contributions of specific mental disorders in the context of different risk time frames to better understand rapidly-developing SA.

## Conclusions

Soldiers with MDD/No-SI are already identified in the health care system and therefore can be offered evidence-based interventions tailored to their risks. The current study findings are important in identifying those at greatest risk, and inform timely and appropriate clinical decisions and interventions. Those who attempt within 30 days of a depression diagnosis include soldiers with five diagnoses: alcohol use disorder or somatoform or dissociative disorder before depression diagnosis, or same-day comorbid bipolar disorder, traumatic stress, or “other” disorder diagnoses. Combat medics and those with less education are also at imminent SA risk. The PARP of 25.2% indicates that if, with appropriate treatment or intervention, risk could be reduced to medium risk level, SAs would be reduced by as much as 25.2%. Future research should examine contributions of treatment and treatment-related factors in altering transition from first depression diagnosis to SA and develop predictive algorithms as clinical assistance tools to better identify soldiers with MDD/No-SI at increased SA risk.

## Electronic supplementary material

Below is the link to the electronic supplementary material.

Supplementary Material 1

## Data Availability

The datasets generated and/or analyzed during the current study are not publicly available, but limited public access to Army STARRS survey data can be requested through the Interuniversity Consortium for Political and Social Research (ICPSR) at the University of Michigan (https://www.icpsr.umich.edu/web/ICPSR/studies/35197).

## List of abbreviations

CI: Confidence interval
DoD: Department of Defense
DoDSER: Department of Defense Suicide Event Report
HADS: Historical Administrative Data Study
MDD: Major depressive disorder
MDD/No-SI: Major depressive disorder with no history of suicidal ideation (diagnosed before or same day of depression diagnosis)
OR: Odds ratio
PARP: Population-attributable risk proportion
PPV: Positive predictive value
PTSD: Posttraumatic stress disorder
SA: Suicide attempt
SI: Suicidal ideation
STARRS: Army Study of Assess Risk and Resilience in Servicemembers
