# Supplementary material for: Predictors of suicide attempt within 30 days of first medically documented major depression diagnosis in U.S. army soldiers with no prior suicidal ideation
Source: BMC Psychiatry. 2023 Jun 2;23:392. doi: 10.1186/s12888-023-04872-z (PMC10239190; doi:10.1186/s12888-023-04872-z)
Supplement: Supplementary file 1 — Supplementary Material 1 [file 12888_2023_4872_MOESM1_ESM.docx]

**SUPPLEMENT**

Predictors of Suicide Attempt within 30 Days of First Medically Documented Major Depression Diagnosis in U.S. Army Soldiers with No Prior Suicidal Ideation

Mash et al.

**Predictors of Suicide Attempt within 30 Days of First Medically Documented Major Depression Diagnosis in U.S. Army Soldiers with No Prior Suicidal Ideation**

**Study Measures**

**Suicide ideation and suicide attempt.** Soldiers with no documented episode of suicidal ideation prior to or on the same day of first documented depression, and those with a suicide attempt within 30 days of their first major depression diagnosis were identified. Soldiers who attempted suicide within 30 days of their suicidal ideation diagnosis were labeled as ‘cases’ and those who did not were ‘controls.’ Classification was accomplished using administrative records from: the DoDSER (Gahm et al., 2012), a DoD-wide surveillance mechanism that aggregates suicidal behavior information via a standardized form completed by medical providers at DoD treatment facilities; and the ICD-9-CM V62.84 and ICD-10-CM R45.851 codes (indicating SI) and ICD-9-CM E950-E958 (indicating self-inflicted poisoning or injury with suicidal intent) and ICD-10-CM T14.91(suicide attempt) codes from the Military Health System Data Repository (MDR), Theater Medical Data Store (TMDS), and TRANSCOM (Transportation Command) Regulating and Command and Control Evacuating System (TRAC^2^ES), which together provide healthcare encounter information from military and civilian treatment facilities, combat operations, and aeromedical evacuations (Table S1, available online at [www.starrs-ls.org/#/list/publications](http://www.starrs-ls.org/#/list/publications)). The E959 code (late effects of a self-inflicted injury) was excluded, as it confounds the temporal relationships between the predictor variables and suicide attempt (Walkup et al., 2012). Records from different data systems were cross-referenced to ensure all cases represent unique soldiers.

**Socio-demographic and service-related characteristics.** Army and DoD administrative personnel records were used to construct socio-demographic (gender, current age, race/ethnicity, education, marital status) and service-related variables (age at Army entry, time in service, deployment status [never, currently, previously], demotion, delayed promotion, and military occupation [combat arms (which includes combat arms and special forces), combat medic, other; Table S2].

**Psychiatric diagnosis.** Administrative medical records identified 26 categories of documented mental disorder diagnoses defined by aggregated ICD-9-CM and ICD-10-CM codes (e.g., attention-deficit/hyperactivity learning disorders [ICD-9-CM codes 314.0-315.9] and ICD-10-CM codes F80.0-F90.0, H93.25, and R48.0]), and ICD-9-CM V codes and ICD-10-CM Z codes for stressors/adversities and marital problems (ICD-9-CM: V40.0-V40.9, V61-V61.9, V62-V62.90, V69.5-V69.9; and ICD-10-CM: Z55.0-Z69.02, Z72.82-Z73.9) and suicidal ideation (ICD-9-CM V62.84 and ICD-10-CM R45.851 codes) (Table S3). Each mental disorder was categorized into two separate time periods, identified by those disorders occurring on the same day as the first major depression diagnosis and those identified as occurring prior to the day of first depression diagnosis. The indicator variables included each of the ICD-9-CM and ICD-10-CM mental disorder diagnostic codes and stressors/adversities and marital problems V and Z codes, respectively. This definition of mental disorder diagnosis detected all soldiers with and without documented, clinically significant, mental health difficulties. In most cases, absence of mental disorder diagnosis indicates significant symptoms were not reported or detected during routine medical care.

| TABLE S1. List and Brief Descriptions of Administrative Data Systems Included in the 2004-2009 Army STARRS Historical Administrative Data Study (HADS). | |
| --- | --- |
|  |  |
| **Database Acronym** | **Description** |
|  |  |
| AFMETS | ARMED FORCES MEDICAL EXAMINER TRACKING SYSTEM (AFMETS): Variables include manner of death and cause of death, including self-inflicted. |
|  |  |
| DCIPS | DEFENSE CASUALTY INFORMATION PROCESSING SYSTEM (DCIPS): Casualty data system for Army, Navy, Air Force, and Marines. It contains information on all casualties including deceased active duty members (and cause of death). |
|  |  |
| DMDC/CTS | DEFENSE MANPOWER DATA CENTER (DMDC) / CONTINGENCY TRACKING SYSTEM (CTS): Collection of activation, mobilization, and deployment data. Provides information to DoD decision makers and includes a CTS Deployment File used for tracking the location of deployed personnel. |
|  |  |
| DMDC/Master Personnel & DMDC/Transaction files | DEFENSE MANPOWER DATA CENTER (DMDC) / MASTER PERSONNEL & TRANSACTION FILES: The Active Duty Master File provides an inventory of all individuals on active duty (excluding reservists on active duty for training) at a point in time. It is a standardized and centralized database of present and past members of the active duty force. Personal data elements include social security number, education level, home of record, date of birth, marital status, number of dependents, race, ethnic group, and name. Military data elements include Service, pay grade, Armed Forces Qualification Test percentile (enlisted only), source of commission (officers only), military primary duty and secondary occupation, Unit Identification Code, months of service, duty location, Estimated Termination of Service date, basic active service date, date of current rank, pay entry base date, foreign language ability, and major command code. |
|  |  |
| DODSER | DEPARTMENT OF DEFENSE SUICIDE EVENT REPORT (DODSER): Provides risk and protective factor information for suicide events. This file contains non-fatal attempts, completed suicides, and suicide ideation cases. |
|  |  |
| MDR | MILITARY HEALTH SYSTEM DATA REPOSITORY (MDR): This database contains information about medical, dental, pharmaceutical, and ancillary claims data for both in network and purchased care as well as both inpatient and outpatient treatment. Data are collected on both Army personnel and their beneficiaries. |
|  |  |
| DMDC/DEERS | DEFENSE MANPOWER DATA CENTER (DMDC) / DEFENSE ENROLLMENT ELIGIBILITY REPORTING SYSTEM (DEERS): A DoD PDR containing personnel data and data related to DoD affiliation, benefits, employment, and pay. |
|  |  |
| TMDS | THEATER MEDICAL DATA STORE (TMDS): Used to track, analyze, view and manage Soldier medical treatment information recorded in the theater of operations. Features of TMDS: accessibility and visibility of service members' deployed medical records, outpatient and inpatient treatment records created in theater facilities, treatment records from other applications, reports on movement of patients, patient status and injury/illnesses. |
|  |  |
| TRAC2ES | TRANSCOM REGULATING AND COMMAND AND CONTROL EVACUATION SYSTEM (TRAC2ES): A tracking system for all medical transfers across the world for all DOD services. |

| **TABLE S2. List of Military Occupational Specialties (MOS) in the 2004-2009 Army STARRS Historical Administrative Data Study (HADS) that were Categorized at Combat Arms, Special Forces, and Combat Medic** | | |
| --- | --- | --- |
|  | | |
| **Combat Arms** | | |
|  | E11B | Infantryman |
|  | E11C | Indirect Fire Infantryman |
|  | E11Z | Infantry Senior Sergeant |
|  | E13B | Cannon Crewmember |
|  | E13E | Cannon Fire Direction Specialist |
|  | E13F | Fire Support Specialist |
|  | E13S | Field Artillery Surveyor |
|  | E19D | Cavalry Scout |
|  | E19K | M1 Armor Crewman |
|  | E19Z | Armor Senior Sergeant |
|  |  |  |
| **Special Forces** | | |
|  | E00D | Special Duty Assignment |
|  | E18B | Special Forces Weapons Sergeant |
|  | E18C | Special Forces Engineer Sergeant |
|  | E18E | Special Forces Communications Sergeant |
|  | E18F | Special Forces Assistant Operations and Intelligence Sergeant |
|  | E18Z | Special Forces Senior Sergeant |
|  |  |  |
| **Combat Medic** | | |
|  | E68W | Health Care Specialist |
|  | E91W | Health Care Specialist |
|  | | |

| **Table S3. ICD-9-CM** **(CDC, 2013)**  **and ICD-10-CM** **(CDC, 2019) codes** **used to identify mental diagnoses and behavioral stressors** | | |
| --- | --- | --- |
|  |  |  |
| **Diagnoses** | **ICD-9-CM Codes** | **ICD-10-CM Codes** |
| **I. Mental diagnoses** |  |  |
| Adjustment disorders | 309.29, 309.3, 309.4, 309.82, 309.83, 309.89, 309.9 | F43.20, F43.24, F43.25, F43.29, F43.8, F43.9 |
|  |  |  |
| Dysthymic disorder/Neurasthenia/Depression NOS | 296.82, 296.90, 296.99, 300.4, 300.5, 309.0, 309.1, 313.1 | F32.8, F32.81, F32.89, F33.8, F34, F34.1, F34.8, F34.81, F34.89, F34.9, F39, F43.21 |
|  |  |  |
| Major depression | 296.20, 296.21, 296.22, 296.23, 296.24, 296.25, 296.26, 296.30, 296.31, 296.32, 296.33, 296.34, 296.35, 296.36, 311 | F32, F32.0, F32.1, F32.2, F32.3, F32.4, F32.5, F32.9, F33, F33.0, F33.1, F33.2, F33.3, F33.4, F33.40, F33.41, F33.42, F33.9 |
|  |  |  |
| Bipolar disorder | 296.00, 296.01, 296.02, 296.03, 296.04, 296.05, 296.06, 296.10, 296.11, 296.12, 296.13, 296.14, 296.15, 296.16, 296.40, 296.41, 296.42, 296.43, 296.44, 296.45, 296.46, 296.50, 296.51, 296.52, 296.53, 296.54, 296.55, 296.56, 296.60, 296.61, 296.62, 296.63, 296.64, 296.65, 296.66, 296.7, 296.80, 296.81, 296.89, 301.13 | F30, F30.1, F30.10, F30.11, F30.12, F30.13, F30.2, F30.3, F30.4, F30.8, F30.9, F31, F31.0, F31.1, F31.10, F31.11, F31.12, F31.13, F31.2, F31.3, F31.30, F31.31, F31.32, F31.4, F31.5, F31.6, F31.60, F31.61, F31.62, F31.63, F31.64, F31.7, F31.70, F31.71, F31.72, F31.73, F31.74, F31.75, F31.76, F31.77, F31.78, F31.8, F31.81, F31.89, F31.9, F34.0 |
|  |  |  |
| Anxiety state/anxiety disorder | 300.00, 300.01, 300.02, 300.09, 300.20, 300.21, 300.22, 300.23, 300.29, 300.3, 309.21, 309.24, 309.28, 313.0, 313.21, 313.22, 313.23 | F40, F40.0, F40.00, F40.01, F40.02, F40.1, F40.10, F40.11, F40.2, F40.21, F40.210, F40.218, F40.22, F40.220, F40.228, F40.23, F40.230, F40.231, F40.232, F40.233, F40.24, F40.240, F40.241, F40.242, F40.243, F40.248, F40.29, F40.290, F40.291, F40.298. F40.8, F40.9, F41, F41.0, F41.1, F41.3, F41.8, F41.9, F42, F42.2, F42.3, F42.4, F42.8, F42.9, F43.2, F43.22, F43.23, F93, F93.0, F94.0 |
|  |  |  |
| PTSD | 309.81 | F43.1, F43.10, F43.11, F43.12 |
|  |  |  |
| Traumatic stress | 308.0, 308.1, 308.2, 308.3, 308.4, 308.9 | F43, F43.0, R45.7 |
|  |  |  |
| ADHD/learning disorders | 314.00, 314.01, 314.1, 314.2, 314.8, 314.9, 315.00, 315.01, 315.02, 315.09, 315.1, 315.2, 315.31, 315.32, 315.34, 315.39, 315.4, 315.5, 315.8, 315.9 | F80, F80.0, F80.1, F80.2, F80.4, F80.8, F80.81, F80.82, F80.89, F80.9, F81, F81.0, F81.2, F81.8, F81.81, F81.89, F81.9, F82, F88, F89, F90, F90.0, F90.1, F90.2, F90.8, F90.9, H93.25, R48.0 |
|  |  |  |
| Conduct disorder/oppositional defiant disorder | 301.7, 312.4, 312.81, 312.82, 312.89, 312.9, 313.81, V62.83 | F60.2, F91, F91.0, F91.1, F91.2, F91.3, F91.8, F91.9, Z69.021, Z69.82 |
|  |  |  |
| Eating disorders | 307.1, 307.50, 307.51, 307.59 | F50, F50.0, F50.00, F50.01, F50.02, F50.2, F50.8, F50.81, F50.82, F50.89, F50.9, F98.29 |
|  |  |  |
| Other impulse control disorders | 312.00, 312.01, 312.02, 312.03, 312.10, 312.11, 312.12, 312.13, 312.20, 312.21, 312,22, 312,23, 312.30, 312.31, 312.32, 312.33, 312.34, 312.35, 312.39 | F63, F63.0, F63.1, F63.2, F63.3, F63.8, F63.81, F63.89, F63.9 |
|  |  |  |
| Alcohol-induced mental disorders/dependence/abuse | 291.0, 291.1, 291.2, 291.3, 291.4, 291.5, 291.81, 291.82, 291.89, 291.9, 303.00, 303.01, 303.02, 303.03, 303.90, 303.91, 303.92, 303.93, 305.00, 305.01, 305.02, 305.03 | F10, F10.1, F10.10, F10.11, F10.12, F10.120, F10.121, F10.129, F10.14, F10.15, F10.150, F10.151, F10.159, F10.18, F10.180, F10.181, F10.182, F10.188, F10.19, F10.2, F10.20, F10.21, F10.22, F10.220, F10.221, F10.229, F10.23, F10.230, F10.231, F10.232, F10.239, F10.24, F10.25, F10.250, F10.251, F10.259, F10.26, F10.27, F10.28, F10.280, F10.281, F10.282, F10.288, F10.29, F10.9, F10.92, F10.920, F10.921, F10.929, F10.94, F10.95, F10.950, F10.951, F10.959, F10.96, F10.97, F10.98, F10.980, F10.981, F10.982, F10.988, F10.99 |
|  |  |  |
| Drug-induced mental disorders | 292.0, 292.11, 292.12, 292.2, 292.81, 292.82, 292.83, 292.84, 292.85, 292.89, 292.9 | F11.121, F11.122, F11.14, F11.15, F11.150, F11.151, F11.159, F11.18, F11.181, F11.182, F11.188, F11.19, F11.221, F11.222, F11.24, F11.25, F11.250, F11.251, F11.259, F11.28, F11.281, F11.282, F11.288, F11.29, F11.921, F11.922, F11.94, F11.95, F11.950, F11.951, F11.959, F11.98, F11.981, F11.982, F11.988, F11.99, F12.121, F12.122, F12.15, F12.150, F12.151, F12.159, F12.18, F12.180, F12.188, F12.19, F12.221, F12.222, F12.25, F12.250, F12.251, F12.28, F12.280, F12.288, F12.29, F12.921, F12.922, F12.95, F12.950, F12.951, F12.959, F12.98, F12.980, F12.988, F12.99, F13.121, F13.14, F13.15, F13.150, F13.151, F13.159, F13.18, F13.180, F13.181, F13.182, F13.188, F13.19, F13.221, F13.23, F13.231, F13.232, F13.24, F13.25, F13.250, F13.251, F13.26, F13.27, F13.28, F13.280, F13.281, F13.282, F13.288, F13.29, F13.921, F13.931, F13.932, F13.94, F13.95, F13.950, F13.951, F13.959, F13.96, F13.97, F13.98, F13.980, F13.981, F13.982, F13.988, F13.99, F14.121, F14.122, F14.14, F14.15, F14.150, F14.151, F14.159, F14.18, F14.180, F14.181, F14.182, F14.188, F14.19, F14.221, F14.222, F14.23, F14.24, F14.25, F14.250, F14.251, F14.28, F14.280, F14.281, F14.282, F14.288, F14.29, F14.921, F14.922, F14.94, F14.95, F14.950, F14951, F14.959, F14.98, F14.980, F14.981, F14.982, F14.988, F14.99, F15.121, F15.122, F15.14, F15.15, F15.150, F15.151, F15.159, F15.18, F15.180, F15.181, F15.182, F15.188, F15.19, F15.221, F15.222, F15.24, F15.25, F15.520, F15.251, F15.259, F15.28, F15.280, F15.281, F15.282, F15.288, F15.29, F15.921, F15.922, F15.93, F15.94, F15.95, F15.950, F15.951, F15.959, F15.98, F15.980, F15.981, F15.982, F15.988, F15.99, F16.121, F16.122, F16.14, F16.15, F16.150, F16.151, F16.159, F16.18, F16.180, F16.183, F16.188, F16.19, F16.221, F16.24, F16.25, F16.250, F16.251, F16.259, F16.28, F16.280, F16.283, F16.288, F16.29, F16.921, F16.94, F16.95, F16.950, F16.951, F16.959, F16.98, F16.980, F16.983, F16.988, F16.99, F18.121, F18.14, F18.15, F18.150, F18.151, F18.159, F18.17, F18.18, F18.180, F18.188, F18.19, F18.221, F18.24, F18.25, F18.250, F18.251, F18.259, F18.27, F18.28, F18.280, F18.288, F18.29, F18.921, F18.94, F18.95, F18.950, F18.951, F18.959, F18.97, F18.98, F18.980, F18.988, F18.99, F19.121, F19.122, F19.14, F19.15, F19.150, F19.151, F19.159, F19.16, F19.17, F19.18, F19.180, F19.181, F19.182, F19.188, F19.19, F19.221, F19.222, F19.231, F19.232, F19.24, F19.25, F19.250, F19.251, F19.259, F19.26, F19.27, F19.28, F19.280, F19.281, F19.282, F19.288, F19.29, F19.921, F19.922, F19.931, F19.932, F19.94, F19.95, F19.950, F19.951, F19.959, F19.96, F19.97, F19.98, F19.980, F19.981, F19.982, F19.988, F19.99 |
|  |  |  |
| Drug abuse without dependence | 305.20, 305.21, 305.22, 305.23, 305.30, 305.31, 305.32, 305.33, 305.40, 305.41, 305.42, 305.43, 305.50, 305.51, 305.52, 305.53, 305.60, 305.61, 305.62, 305.63, 305.70, 305.71, 305.72, 305.73, 305.80, 305.81, 305.82, 305.83, 305.90, 305.91, 305.92, 305.93 | F11, F11.1, F11.10, F11.11, F11.12, F11.120, F11.129, F11.9, F11.90, F11.92, F11.920, F11.929, F11.93, F12, F12.1, F12.10, F12.11, F12.12, F12.120, F12.129, F12.9, F12.90, F12.92, F12.920, F12.929, F12.93, F13, F13.1, F13.10, F13.11, F13.12, F13.120, F13.129, F13.9, F13.90, F13.92, F13.920, F13.929, F13.93, F13.930, F13.939, F14, F14.1, F14.10, F14.11, F14.12, F14.120, F14.129, F14.9, F14.90, F14.92, F14.920, F14.929, F15, F15.1, F15.10,F15.11, F15.12, F15.120, F15.129, F15.9, F15.90, F15.92, F15.920, F15.929, F16, F16.1, F16.10, F16.11, F16.12, F16.120, F16.129, F16.9, F16.90, F16.92, F16.920, F16.929, F17.208, F17.218, F17.219, F17.228, F17.298, F17.299, F18, F18.1, F18.10, F18.11, F18.12, F18.120, F18.129, F18.9, F18.90, F18.92, F18.920, F18.929, F19, F19.1, F19.10, F19.11, F19.12, F19.120, F19.129, F19.9, F19.90, F19.92, F19.920, F19.929, F19.93, F19.930, F19.939, F55, F55.0, F55.1, F55.2, F55.3, F55.4, F55.8 |
|  |  |  |
| Drug dependence | 304.00, 304.01, 304.02, 304.03, 304.10, 304.11, 304.12, 304.13, 304.20, 304.21, 304.22, 304.23, 304.30, 304.31, 304.32, 304.33, 304.40, 304.41, 304.42, 304.43, 304.50, 304.51, 304.52, 304.53, 304.60, 304.61, 304.62, 304.63, 304.70, 304.71, 304.72, 304.73, 304.80, 304.81, 304.82, 304.83, 304.90, 304.91, 304.92, 304.93, | F11.2, F11.20, F11.21, F11.22, F11.220, F11.229, F11.23, F12.2, F12.20, F12.21, F12.22, F12.220, F12.229, F12.23, F12.259, F13.2, F13.20, F13.21, F13.22, F13.220, F13.229, F13.230, F13.239, F13.259, F14.2, F14.20, F14.21, F14.22, F14.220, F14.229, F14.259, F15.2, F15.20, F15.21, F15.22, F15.220, F15.229, F15.23, F16.2, F16.20, F16.21, F16.22, F16.220, F16.229, F18.2, F18.20, F18.21, F18.22, F18.220, F18.229, F19.2, F19.20, F19.21, F19.22, 19.220, F19.229, F19.23, F19.230, F19.239 |
|  |  |  |
| Tobacco use disorder | 305.1 | F17, F17.2, F17.20, F17.200, F17.201, F17.203, F17.209, F17.21, F17.210, F17.211, F17.213, F17.22, F17.220, F17.221, F17.223, F17.229, F17.29, F17.290, F17.291, F17.293, Z72.0 |
|  |  |  |
| Personality disorders | 301.0, 301.10, 301.11, 301.12, 301.20, 301.21, 301.22, 301.3, 301.4, 301.50, 301.51, 301.59, 301.6, 301.81, 301.82, 301.83, 301.84, 301.89, 301.9 | F21, F60, F60.0, F60.1, F60.3, F60.4, F60.5, F60.6, F60.7, F60.8, F60.81, F60.89, F60.9, F68.10, F68.12, F68.A, F69 |
|  |  |  |
| Non-affective psychosis | 295.00, 295.01, 295.02, 295.03, 295.04, 295.05, 295.10, 295.11, 295.12, 295.13, 295.14, 295.15, 295.20, 295.21, 295.22, 295.23, 295.24, 295.25, 295.30, 295.31, 295.32, 295.33, 295.34, 295.35, 295.40, 295.41, 295.42, 295.43, 295.44, 295.45, 295.50, 295.51, 295.52, 295.53, 295.54, 295.55, 295.60, 295.61, 295.62, 295.63, 295.64, 295.65, 295.70, 295.71, 295.72, 295.73, 295.74, 295.75, 295.80, 295.81, 295.82, 295.83, 295.84, 295.85, 295.90, 295.91, 295.92, 295.93, 295.94, 295.95, 297.0, 297.1, 297.2, 297.3, 297.8, 297.9, 298.0, 298.1, 298.2, 298.3, 298.4, 298.8, 298.9 | F20, F20.0, F20.1, F20.2, F20.3, F20.5, F20.8, F20.81, F20.89, F20.9, F22, F23, F24, F25, F25.0, F25.1, F25.8, F25.9, F28, F29 |
|  |  |  |
| Somatoform/dissociative disorders | 300.10, 300.11, 300.12, 300.13, 300.14, 300.15, 300.16, 300.19, 300.6, 300.7, 300.81, 300.82, 300.89, 306.0, 306.1, 306.2, 306.3, 306.4, 306.50, 306.51, 306.52, 306.53, 306.59, 306.6, 306.7, 306.8, 306.9, 307.54, 307.80, 307.81, 307.89 | F44, F44.0, F44.1, F44.2, F44.4, F44.5, F44.6, F44.7, F44.8, F44.81, F44.89, F44.9, F45, F45.0, F45.1, F45.2, F45.20, F45.21, F45.22, F45.29, F45.4, F45.41, F45.42, F45.8, F45.9, F48.1, F48.2, F48.8, F52.5, F59, F68, F68.1, F68.11, F68.13, F68.8, G44.209 |
|  |  |  |
| Organic mental disorders | 290.0, 290.10, 290.11, 290.12, 290.13, 290.20, 290.21, 290.3, 290.40, 290.41, 290.42, 290.43, 290.8, 290.9, 293.0, 293.1, 293.81, 293.82, 293.83, 293.84, 293.89, 293.9, 294.0, 294.10, 294.11, 294.20, 294.21, 294.8, 294.9, 307.20, 307.21, 307.22, 307.23, 307.3, 310.0, 310.81, 310.89, 310.9, 317, 318.0, 318.1, 318.2, 319 | F01, F01.5, F01.50, F01.51, F02, F02.8, F02.80, F02.81, F03, F03.9, F03.90, F03.91, F04, F05, F06, F06.0, F06.1, F06.2, F06.3, F06.30, F06.31, F06.32, F06.33, F06.34, F06.4, F06.8, F07, F07.0, F07.8, F07.89, F07.9, F09, F53, F53.0, F53.1, F70, F71, F72, F73, F78, F79, F95, F95.0, F95.1, F95.2, F95.8, F95.9, F98.4, R41.83 |
|  |  |  |
| Sexual disorders | 302.0, 302.1, 302.2, 302.3, 302.4, 302.50, 302.51, 302.52, 302.53, 302.6, 302.70, 302.71, 302.72, 302.73, 302.74, 302.75, 302.76, 302.79, 302.81, 302.82, 302.83, 302.84, 302.85, 302.89, 302.9 | F52, F52.0, F52.1, F52.2, F52.21, F52.22, F52.3, F52,31, F52.32, F52.4, F52.6, F52.8, F52.9, F64, F64.0, F64,1, F64.2, F64,8, F64,9, F65, F65.0, F65.1, F65.2, F65.3, F65,4, F65.5, F65.50, F65.51, F65.52, F65.8, F65.81, F65.89, F65.9, F66, R37, Z87.890 |
|  |  |  |
| Sleep disorders | 307.40, 307.41, 307.42, 307.43, 307.44, 307.45, 307.46, 307.47, 307.48, 307.49 | F51, F51.0, F51.01, F51.02, F51.03, F51.04, F51.05, F51.09, F51.1, F51.11, F51.12, F51.13, F51.19, F51.3, F51.4, F51.5, F51.8, F51.9 |
|  |  |  |
| Other mental disorders/mental illness | 299.00, 299.01, 299.10, 299.11, 299.80, 299.81, 299.90, 299.91, 300.9, 307.0, 307.52, 307.53, 307.6, 307.7, 307.9, 309.22, 310.1, 313.3, 313.82, 313.89, 313.9, 316 | F48, F48.9, F54, F84, F84.0, F84.2, F84.3, F84.5, F84.8, F84.9, F93.8, F93.9, F94, F94.1, F94.2, F94.8, F94.9, F98, F98.0, F98.1, F98.2, F98.21, F98.3, F98.5, F98.8, F98.9, F99, R45.1, R45.2, R45.5, R45.6, R45.81, R45.82 |
| Postconcussion Syndrome | 310.2 | F07.81 |
|  |  |  |
| **II. Suicidal Ideation & V / Z / R Stressor Codes** |  |  |
|  |  |  |
| Suicidal ideation | V62.84 | R45.851 |
|  |  |  |
| Indicator of impulsivity and risky behavior | V69.2, V69.3 | Z72.51, Z72.52, Z72.53, Z72.6 |
|  |  |  |
| Stressors/adversities & marital problems | V40.0, V40.1, V40.2, V40.9, V61.01, V61.02, V61.03, V61.04, V61.05, V61.06, V61.07, V61.08, V61.09, V61.10, V61.11, V61.12, V61.20, V61.21, V61.22, V61.23, V61.24, V61.25, V61.29, V61.3, V61.41, V61.42, V61.49, V61.8, V61.9, V62.0, V62.1, V62.21, V62.22, V62.29, V62.3, V62.4, V62.5, V62.81, V62.82, V62.89, V62.9, V69.4, V69.5, V69.9 | Z55.0, Z55.1, Z55.2, Z55.3, Z55.4, Z55.8, Z55.9, Z56.0, Z56.1, Z56.2, Z56.3, Z56.4, Z56.5, Z56.6, Z56.81, Z56.89, Z56.9, Z57.0, Z57.1, Z57.2, Z57.31, Z57.39, Z57.4, Z57.5, Z57.6, Z57.7, Z57.8, Z57.9, Z60.0, Z60.3, Z60.4, Z60.5, Z60.8, Z60.9, Z62.0, Z62.1, Z62.22, Z62.29, Z62.3, Z62.6, Z62.810, Z62.811, Z62.812, Z62.819, Z62.820, Z62.821, Z62.822, Z62.890, Z62.891, Z62.898, Z62.9, Z63.0, Z63.1, Z63.31, Z63.32, Z63.4, Z63.5, Z63.6, Z63.71, Z63.72, Z63.79, Z63.8, Z63.9, Z64.4, Z65.0, Z65.1, Z65.2, Z65.3, Z65.4, Z65.5, Z65.8, Z65.9, Z69.010, Z69.011, Z69.020, Z69.11, Z69.12, Z72.820, Z72.821, Z72.89, Z72.9, Z73.0, Z73.1, Z73.2, Z73.3, Z73.4, Z73.5, Z73.6, Z73.810, Z73.811, Z73.812, Z73.819, Z73.89, Z73.9 |
|  |  |  |

Abbreviations: ICD-9-CM, International Classification of Diseases, Ninth Revision Clinical Modification; ICD-10-CM, International Classification of Diseases, Tenth Revision Clinical Modification; PTSD, post-traumatic stress disorder; ADHD, attention deficit-hyperactivity disorder.

Centers for Disease Control and Prevention. The International Classification of Diseases, ninth revision, Clinical Modification (ICD-9-CM). 2013; https://www.cdc.gov/nchs/icd/icd9cm.htm. Accessed June 10, 2021.

Centers for Disease Control and Prevention. The International Classification of Diseases, tenth revision, Clinical Modification (ICD-10-CM). 2019; https://www.cdc.gov/nchs/icd/icd10cm.htm. Accessed June 10, 2021.

**TABLE S4. Association of documented psychiatric diagnoses (day of and prior to major depression diagnosis) of active-duty Regular U.S. Army enlisted soldiers with documented suicide attempt within 30 days following initial depression diagnosis**

|  | **Univariate** | | | **Soldiers with Major Depression and No Prior/Same Day Suicidal Ideation** | | | | **Total population**  **(n = 101,046)** |
| --- | --- | --- | --- | --- | --- | --- | --- | --- |
|  |  | | | **Attempted suicide within 30 days^b^**  **(n = 421)** | | **Did not attempt suicide within 30 days^c^**  **(n = 100,625)** | |  |
|  | **OR^d^** | **(95% CI^d^)** | | ***n*** | **%** | ***n*** | **%** | **%** |
| **Psychiatric Diagnoses** |  |  | |  |  |  |  |  |
| **ADHD/ Learning Disorders** |  |  | |  |  |  |  |  |
| Day of depression diagnosis |  |  | |  |  |  |  |  |
| No | 1.0 | – | | 410 | 97.4 | 98,530 | 97.9 | 97.9 |
| Yes | 1.3 | (0.7–2.3) | | 11 | 2.6 | 2,095 | 2.1 | 2.1 |
| χ²_1_ | 0.59 | | |  |  |  |  |  |
| Prior to depression diagnosis |  |  | |  |  |  |  |  |
| No | 1.0 | – | | 403 | 95.7 | 95,622 | 95.0 | 95.0 |
| Yes | 0.9 | (0.5–1.4) | | 18 | 4.3 | 5,003 | 5.0 | 5.0 |
| χ²_1_ | 0.41 | | |  |  |  |  |  |
| **Adjustment Disorder** |  |  | |  |  |  |  |  |
| Day of depression diagnosis |  |  | |  |  |  |  |  |
| No | 1.0 | – | | 395 | 93.8 | 97,506 | 96.9 | 96.9 |
| Yes | 2.1* | (1.4–3.1) | | 26 | 6.2 | 3,119 | 3.1 | 3.1 |
| χ²_1_ | 12.57* | | |  |  |  |  |  |
| Prior to depression diagnosis |  |  | |  |  |  |  |  |
| No | 1.0 | – | | 313 | 74.3 | 75,296 | 74.8 | 74.8 |
| Yes | 1.0 | (0.8–1.3) | | 108 | 25.7 | 25,329 | 25.2 | 25.2 |
| χ²_1_ | 0.07 | | |  |  |  |  |  |
| **Alcohol Use Disorder** |  |  | |  |  |  |  |  |
| Day of depression diagnosis |  |  | |  |  |  |  |  |
| No | 1.0 | – | | 381 | 90.5 | 95,245 | 94.7 | 94.6 |
| Yes | 1.9* | (1.3–2.6) | | 40 | 9.5 | 5,380 | 5.3 | 5.4 |
| χ²_1_ | 13.94* | | |  |  |  |  |  |
| Prior to depression diagnosis |  | | |  |  |  |  |  |
| No | 1.0 | | – | 347 | 82.4 | 87,164 | 86.6 | 86.6 |
| Yes | 1.4* | | (1.1–1.8) | 74 | 17.6 | 13,461 | 13.4 | 13.4 |
| χ²_1_ | 6.41* | | |  |  |  |  |  |
| **Anxiety Disorder** |  |  | |  |  |  |  |  |
| Day of depression diagnosis |  |  | |  |  |  |  |  |
| No | 1.0 | – | | 328 | 77.9 | 82,633 | 82.1 | 82.1 |
| Yes | 1.3* | (1.0–1.7) | | 93 | 22.1 | 17,992 | 17.9 | 17.9 |
| χ²_1_ | 5.21* | | |  |  |  |  |  |
| Prior to depression diagnosis |  |  | |  |  |  |  |  |
| No | 1.0 | – | | 279 | 66.3 | 59,155 | 58.8 | 58.8 |
| Yes | 0.7* | (0.6–0.9) | | 142 | 33.7 | 41,470 | 41.2 | 41.2 |
| χ²_1_ | 9.24* | | |  |  |  |  |  |
| **Bipolar Disorder** |  |  | |  |  |  |  |  |
| Day of depression diagnosis |  |  | |  |  |  |  |  |
| No | 1.0 | – | | 412 | 97.9 | 100,318 | 99.7 | 99.7 |
| Yes | 7.1* | (3.6–13.8) | | 9 | 2.1 | 307 | 0.3 | 0.3 |
| χ²_1_ | 32.71* | | |  |  |  |  |  |
| Prior to depression diagnosis |  |  | |  |  |  |  |  |
| No | 1.0 | – | | 417 | 99.0 | 99,770 | 99.2 | 99.1 |
| Yes | 1.1 | (0.4–3.0) | | 4 | 1.0 | 855 | 0.8 | .9 |
| χ²_1_ | 0.05 | | |  |  |  |  |  |
| **Conduct Disorder/ Oppositional Defiant Disorder** |  |  | |  |  |  |  |  |
| Day of depression diagnosis |  |  | |  |  |  |  |  |
| No | 1.0 | – | | 419 | 99.5 | 100,494 | 99.9 | 99.9 |
| Yes | 3.7 | (0.9–14.8) | | 2 | 0.5 | 131 | 0.1 | 0.1 |
| χ²_1_ | 3.31 | | |  |  |  |  |  |
| Prior to depression diagnosis |  |  | |  |  |  |  |  |
| No | 1.0 | – | | 419 | 99.5 | 100,225 | 99.6 | 99.6 |
| Yes | 1.2 | (0.3–4.8) | | 2 | 0.5 | 400 | 0.4 | 0.4 |
| χ²_1_ | 0.06 | | |  |  |  |  |  |
| **Dysthymic Disorder/**  **Neurasthenia/Depression NOS** |  |  | |  |  |  |  |  |
| Day of depression diagnosis |  |  | |  |  |  |  |  |
| No | 1.0 | – | | 383 | 91.0 | 96,675 | 96.1 | 96.1 |
| Yes | 2.4* | (1.7–3.4) | | 38 | 9.0 | 3,950 | 3.9 | 3.9 |
| χ²_1_ | 26.87* | | |  |  |  |  |  |
| Prior to depression diagnosis |  |  | |  |  |  |  |  |
| No | 1.0 | – | | 313 | 74.3 | 75,855 | 75.4 | 75.4 |
| Yes | 1.1 | (0.9–1.3) | | 108 | 25.7 | 24,770 | 24.6 | 24.6 |
| χ²_1_ | 0.30 | | |  |  |  |  |  |
| **Eating Disorders** |  |  | |  |  |  |  |  |
| Day of depression diagnosis |  |  | |  |  |  |  |  |
| No | 1.0 | – | | 421 | 100.00 | 100,485 | 99.9 | 99.9 |
| Yes | – | – | | 0 | 0.00 | 140 | 0.1 | 0.1 |
| χ²_1_ | 0.003 | | |  |  |  |  |  |
| Prior to depression diagnosis |  |  | |  |  |  |  |  |
| No | 1.0 | – | | 420 | 99.8 | 100,389 | 99.8 | 99.8 |
| Yes | 1.0 | (0.1–7.3) | | 1 | 0.2 | 236 | 0.2 | 0.2 |
| χ²_1_ | 0.00 | | |  |  |  |  |  |
| **Stressors/Adversities & Marital Problems** |  |  | |  |  |  |  |  |
| Day of depression diagnosis |  |  | |  |  |  |  |  |
| No | 1.0 | – | | 361 | 85.7 | 89,794 | 89.2 | 89.2 |
| Yes | 1.4* | (1.0–1.8) | | 60 | 14.3 | 10,831 | 10.8 | 10.8 |
| χ²_1_ | 5.32* | | |  |  |  |  |  |
| Prior to depression diagnosis |  |  | |  |  |  |  |  |
| No | 1.0 | – | | 264 | 62.7 | 53,682 | 53.3 | 53.4 |
| Yes | 0.7* | (0.6–0.8) | | 157 | 37.3 | 46,943 | 46.7 | 46.6 |
| χ²_1_ | 14.08* | | |  |  |  |  |  |
| **Non-Affective Psychosis** |  |  | |  |  |  |  |  |
| Day of depression diagnosis |  |  | |  |  |  |  |  |
| No | 1.0 | – | | 412 | 97.9 | 99,992 | 99.4 | 99.4 |
| Yes | 3.5* | (1.8–6.7) | | 9 | 0.2 | 633 | 0.6 | 0.6 |
| χ²_1_ | 13.39* | | |  |  |  |  |  |
| Prior to depression diagnosis |  |  | |  |  |  |  |  |
| No | 1.0 | – | | 420 | 99.8 | 99,684 | 99.1 | 99.1 |
| Yes | 0.3 | (0.0–1.8) | | 1 | 0.2 | 941 | 0.9 | 0.9 |
| χ²_1_ | 1.90 | | |  |  |  |  |  |
| **Organic Mental Disorders** |  |  | |  |  |  |  |  |
| Day of depression diagnosis |  |  | |  |  |  |  |  |
| No | 1.0 | – | | 417 | 99.0 | 100,006 | 99.4 | 99.4 |
| Yes | 1.5 | (0.6–4.1) | | 4 | 1.0 | 619 | 0.6 | 0.6 |
| χ²_1_ | 0.74 | | |  |  |  |  |  |
| Prior to depression diagnosis |  |  | |  |  |  |  |  |
| No | 1.0 | – | | 411 | 97.6 | 97,237 | 96.6 | 96.6 |
| Yes | 0.7 | (0.4–1.3) | | 10 | 2.4 | 3,388 | 3.4 | 3.4 |
| χ²_1_ | 1.25 | | |  |  |  |  |  |
| **Other Disorder^e^** |  |  | |  |  |  |  |  |
| Day of depression diagnosis |  |  | |  |  |  |  |  |
| No | 1.0 | – | | 384 | 91.2 | 99,693 | 99.1 | 99.0 |
| Yes | 10.3* | (7.3–14.5) | | 37 | 8.8 | 932 | 0.9 | 1.0 |
| χ²_1_ | 176.84* | | |  |  |  |  |  |
| Prior to depression diagnosis |  |  | |  |  |  |  |  |
| No | 1.0 | – | | 406 | 96.4 | 97,414 | 96.8 | 96.8 |
| Yes | 1.1 | (0.7–1.9) | | 15 | 3.6 | 3,211 | 3.2 | 3.2 |
| χ²_1_ | 0.19 | | |  |  |  |  |  |
| **Other Impulse Control Disorders** |  |  | |  |  |  |  |  |
| Day of depression diagnosis |  |  | |  |  |  |  |  |
| No | 1.0 | – | | 418 | 99.3 | 100,207 | 99.6 | 99.6 |
| Yes | 1.7 | (0.5–5.4) | | 3 | 0.7 | 418 | 0.4 | 0.4 |
| χ²_1_ | 0.86 | | |  |  |  |  |  |
| Prior to depression diagnosis |  |  | |  |  |  |  |  |
| No | 1.0 | – | | 418 | 99.3 | 99,385 | 98.8 | 98.8 |
| Yes | 0.6 | (0.2–1.8) | | 3 | 0.7 | 1,240 | 1.2 | 1.2 |
| χ²_1_ | 0.91 | | |  |  |  |  |  |
| **Personality Disorders** |  |  | |  |  |  |  |  |
| Day of depression diagnosis |  |  | |  |  |  |  |  |
| No | 1.0 | – | | 412 | 97.9 | 100,115 | 99.5 | 99.5 |
| Yes | 4.3* | (2.2–8.3) | | 9 | 0.2 | 510 | 0.5 | 0.5 |
| χ²_1_ | 18.28* | | |  |  |  |  |  |
| Prior to depression diagnosis |  |  | |  |  |  |  |  |
| No | 1.0 | – | | 412 | 97.9 | 99,434 | 98.8 | 98.8 |
| Yes | 1.8 | (0.9–3.5) | | 9 | 2.1 | 1,191 | 1.2 | 1.2 |
| χ²_1_ | 3.12 | | |  |  |  |  |  |
| **Sexual Disorders** |  |  | |  |  |  |  |  |
| Day of depression diagnosis |  |  | |  |  |  |  |  |
| No | 1.0 | – | | 420 | 99.8 | 100,249 | 99.6 | 99.6 |
| Yes | 0.6 | (0.1–4.5) | | 1 | 0.2 | 376 | 0.4 | 0.4 |
| χ²_1_ | 0.21 | | |  |  |  |  |  |
| Prior to depression diagnosis |  |  | |  |  |  |  |  |
| No | 1.0 | – | | 417 | 99.0 | 95,954 | 95.4 | 95.4 |
| Yes | 0.2* | (0.1–0.5) | | 4 | 1.0 | 4,671 | 4.6 | 4.6 |
| χ²_1_ | 10.38* | | |  |  |  |  |  |
| **Sleep Disorders** |  |  | |  |  |  |  |  |
| Day of depression diagnosis |  |  | |  |  |  |  |  |
| No | 1.0 | – | | 417 | 99.0 | 97,597 | 97.0 | 97.0 |
| Yes | 0.3* | (0.1–0.8) | | 4 | 1.0 | 3,028 | 3.0 | 3.0 |
| χ²_1_ | 5.49* | | |  |  |  |  |  |
| Prior to depression diagnosis |  |  | |  |  |  |  |  |
| No | 1.0 | – | | 369 | 87.6 | 84,515 | 84.0 | 84.0 |
| Yes | 0.7* | (0.6–1.0) | | 52 | 12.4 | 16,110 | 16.0 | 16.0 |
| χ²_1_ | 4.05* | | |  |  |  |  |  |
| **Somatoform/Dissociative Disorders** |  |  | |  |  |  |  |  |
| Day of depression diagnosis |  |  | |  |  |  |  |  |
| No | 1.0 | – | | 420 | 99.8 | 100,187 | 99.6 | 99.6 |
| Yes | 0.5 | (0.1–3.9) | | 1 | 0.2 | 438 | 0.4 | 0.4 |
| χ²_1_ | 0.37 | | |  |  |  |  |  |
| Prior to depression diagnosis |  |  | |  |  |  |  |  |
| No | 1.0 | – | | 404 | 96.0 | 96,857 | 96.3 | 96.3 |
| Yes | 1.1 | (0.7–1.8) | | 17 | 4.0 | 3,768 | 3.7 | 3.7 |
| χ²_1_ | 0.10 | | |  |  |  |  |  |
| **TBI/Postconcussion Syndrome** |  |  | |  |  |  |  |  |
| Day of depression diagnosis |  |  | |  |  |  |  |  |
| No | 1.0 | – | | 421 | 100.0 | 100,275 | 99.7 | 99.7 |
| Yes | – | – | | 0 | 0.0 | 350 | 0.3 | 0.3 |
| χ²_1_ | 0.003 | | |  |  |  |  |  |
| Prior to depression diagnosis |  |  | |  |  |  |  |  |
| No | 1.0 | – | | 405 | 96.2 | 97,121 | 96.5 | 96.5 |
| Yes | 1.1 | (0.7–1.8) | | 16 | 3.8 | 3,504 | 3.5 | 3.5 |
| χ²_1_ | 0.12 | | |  |  |  |  |  |
| **Tobacco Use Disorder** |  |  | |  |  |  |  |  |
| Day of depression diagnosis |  |  | |  |  |  |  |  |
| No | 1.0 | – | | 380 | 90.3 | 94,676 | 94.1 | 94.1 |
| Yes | 1.7* | (1.2–2.4) | | 41 | 9.7 | 5,949 | 5.9 | 5.9 |
| χ²_1_ | 10.82* | | |  |  |  |  |  |
| Prior to depression diagnosis |  |  | |  |  |  |  |  |
| No | 1.0 | – | | 259 | 61.5 | 58,617 | 58.3 | 58.3 |
| Yes | 0.9 | (0.7–1.1) | | 162 | 38.5 | 42,008 | 41.7 | 41.7 |
| χ²_1_ | 1.83 | | |  |  |  |  |  |
| **Traumatic Stress** |  |  | |  |  |  |  |  |
| Day of depression diagnosis |  |  | |  |  |  |  |  |
| No | 1.0 | – | | 410 | 97.4 | 99,808 | 99.2 | 99.2 |
| Yes | 3.3* | (1.8–3.3) | | 11 | 2.6 | 817 | 0.8 | 0.8 |
| χ²_1_ | 14.76* | | |  |  |  |  |  |
| Prior to depression diagnosis |  |  | |  |  |  |  |  |
| No | 1.0 | – | | 399 | 94.8 | 94,849 | 94.3 | 94.3 |
| Yes | 0.9 | (0.6–1.4) | | 22 | 5.2 | 5,776 | 5.7 | 5.7 |
| χ²_1_ | 0.22 | | |  |  |  |  |  |
| **PTSD** |  |  | |  |  |  |  |  |
| Day of depression diagnosis |  |  | |  |  |  |  |  |
| No | 1.0 | – | | 383 | 91.0 | 88,889 | 88.3 | 88.3 |
| Yes | 0.8 | (0.5–1.1) | | 38 | 9.0 | 11,736 | 11.7 | 11.7 |
| χ²_1_ | 2.75 | | |  |  |  |  |  |
| Prior to depression diagnosis |  |  | |  |  |  |  |  |
| No | 1.0 | – | | 378 | 89.8 | 86,023 | 85.5 | 85.5 |
| Yes | 0.7* | (0.5–0.9) | | 43 | 10.2 | 14,602 | 14.5 | 14.5 |
| χ²_1_ | 6.05* | | |  |  |  |  |  |
| **Drug-Induced Mental Disorders** |  |  | |  |  |  |  |  |
| Day of depression diagnosis |  |  | |  |  |  |  |  |
| No | 1.0 | – | | 416 | 98.8 | 100,158 | 99.5 | 99.5 |
| Yes | 2.6* | (1.1–6.2) | | 5 | 1.2 | 467 | 0.5 | 0.5 |
| χ²_1_ | 4.35* | | |  |  |  |  |  |
| Prior to depression diagnosis |  |  | |  |  |  |  |  |
| No | 1.0 | – | | 405 | 96.2 | 98,113 | 97.5 | 97.5 |
| Yes | 1.5 | (0.9–2.5) | | 16 | 3.8 | 2,512 | 2.5 | 2.5 |
| χ²_1_ | 2.83 | | |  |  |  |  |  |
| **Non-Dependent Drug Abuse** |  |  | |  |  |  |  |  |
| Day of depression diagnosis |  |  | |  |  |  |  |  |
| No | 1.0 | – | | 410 | 97.4 | 99,473 | 98.9 | 98.8 |
| Yes | 2.3* | (1.3–4.2) | | 11 | 2.6 | 1,152 | 1.1 | 1.2 |
| χ²_1_ | 7.41* | | |  |  |  |  |  |
| Prior to depression diagnosis |  |  | |  |  |  |  |  |
| No | 1.0 | – | | 401 | 95.2 | 97,298 | 96.7 | 96.7 |
| Yes | 1.5 | (0.9–2.3) | | 20 | 4.8 | 3,327 | 3.3 | 3.3 |
| χ²_1_ | 2.65 | | |  |  |  |  |  |
| **Drug Dependence** |  |  | |  |  |  |  |  |
| Day of depression diagnosis |  |  | |  |  |  |  |  |
| No | 1.0 | – | | 418 | 99.3 | 99,539 | 98.9 | 98.9 |
| Yes | 0.7 | (0.2–2.0) | | 3 | 0.7 | 1,086 | 1.1 | 1.1 |
| χ²_1_ | 0.53 | | |  |  |  |  |  |
| Prior to depression diagnosis |  |  | |  |  |  |  |  |
| No | 1.0 | – | | 411 | 97.6 | 98,367 | 97.8 | 97.8 |
| Yes | 1.1 | (0.6–2.0) | | 10 | 2.4 | 2,258 | 2.2 | 2.2 |
| χ²_1_ | 0.03 | | |  |  |  |  |  |
| **Indicator of Impulsivity & Risky Behavior** |  |  | |  |  |  |  |  |
| Day of depression diagnosis |  |  | |  |  |  |  |  |
| No | 1.0 | – | | 421 | 100.0 | 100,607 | 99.98 | 99.98 |
| Yes | – | – | | 0 | 0.0 | 18 | 0.02 | 0.02 |
| χ²_1_ | 0.002 | | |  |  |  |  |  |
| Prior to depression diagnosis |  |  | |  |  |  |  |  |
| No | 1.0 | – | | 418 | 99.3 | 99,253 | 98.6 | 98.6 |
| Yes | 0.5 | (0.2–1.6) | | 3 | 0.7 | 1,372 | 1.4 | 1.4 |
| χ²_1_ | 1.28 | | |  |  |  |  |  |

^a^Table S3 includes all ICD-9-CM and ICD-10 codes associated with each mental disorder

^b^Soldiers with first-time documented major depression diagnosis who subsequently attempted suicide within the next 30 days of recorded depression diagnosis

^c^Soldiers with first-time documented major depression diagnosis who did not subsequently attempt suicide within the next 30 days

^d^ OR = Odds ratio; CI = Confidence interval

^e^Among soldiers who were diagnosed with a disorder classified as “Other,” 33/37 (89.2%) were identified by the ICD-9-CM code 300.9 (Unspecified nonpsychotic mental disorder)

**p* < .05

**TABLE S5. Multivariate associations of socio-demographic and service-related characteristics in active-duty Regular U.S. Army enlisted soldiers with documented suicide attempt within 30 days following initial major depression diagnosis^a^**

|  | **OR^b^** | **(95% CI^b^)** |
| --- | --- | --- |
|  |  |  |
| **Socio-demographic characteristics** |  |  |
| Gender |  |  |
| Male | 1.0 | – |
| Female | 1.1 | (0.9-1.4) |
| χ²_1_ | 0.64 | |
| Current Age |  |  |
| < 21 | 2.4* | (1.2-4.9) |
| 21-24 | 1.7 | (1.0-3.1) |
| 25-29 | 1.4 | (0.9-2.3) |
| 30-34 | 1.0 | – |
| 35-39 | 0.8 | (0.4-1.6) |
| 40+ | 0.9 | (0.4-2.1) |
| χ²_5_ | 7.75 | |
| Race/Ethnicity |  |  |
| White | 1.0 | – |
| Black | 1.0 | (0.8-1.3) |
| Hispanic | 1.1 | (0.8-1.4) |
| Asian | 0.5 | (0.3-1.1) |
| Other | 1.1 | (0.4-2.6) |
| χ²_3_ | 3.48 | |
| Education |  |  |
| < High school | 1.5* | (1.2-1.9) |
| High school | 1.0 | – |
| Some college | 0.7 | (0.3-1.5) |
| > College | 0.9 | (0.5-1.7) |
| χ²_3_ | 13.18* | |
| Marital Status |  |  |
| Never married | 1.0 | – |
| Currently married | 0.7* | (0.6-0.9) |
| Previously married | 0.8 | (0.5-1.5) |
| χ²_2_ | 8.13* | |
| **Service-related Characteristics** |  | |
| Age at Army Entry |  |  |
| < 21 | 0.9 | (0.7-1.2) |
| 21-24 | 1.0 | – |
| 25+ | 0.9 | (0.6-1.5) |
| χ²_2_ | 0.43 | |
| Time in Service |  | |
| 1-2 years | 1.5 | (0.9-2.7) |
| 3-4 years | 1.2 | (0.8-1.6) |
| 5-10 years | 1.0 | – |
| > 10 years | 0.4* | (0.2-0.7) |
| χ²_3_ | 10.49* | |
| Deployment Status |  | |
| Never | 1.0 | – |
| Current | 1.4 | (0.9-2.0) |
| Previous | 1.0 | (0.8-1.4) |
| χ²_2_ | 3.03 | |
| Demotion |  | |
| Past year | 1.4 | (0.9-2.3) |
| Before past year | 1.5* | (1.0-2.1) |
| Never demoted | 1.0 | – |
| χ²_2_ | 5.71 | |
| Delayed Promotion |  | |
| On schedule | 1.0 | – |
| Late: < 2 months | 0.6 | (0.2-1.6) |
| Late: > 2 months | 0.8 | (0.4-1.3) |
| Not relevant because of rank^d^ | 0.8 | (0.5-1.3) |
| χ²_3_ | 1.92 | |
| Military Occupational Specialty (MOS) |  | |
| Combat arms^e^ | 1.4* | (1.1-1.7) |
| Combat medics | 1.5* | (1.0-2.1) |
| Other MOS | 1.0 | – |
| χ²_2_ | 9.59* | |

^a^Multivariate model was adjusted for socio-demographics (gender, current age, race, education, and marital status) and service-related characteristics.

^b^OR = Odds ratio; CI = Confidence interval

^c^< High School includes: General Educational Development credential (GED), home study diploma, occupational program certificate, correspondence school diploma, high school certificate of attendance, adult education diploma, and other non-traditional high school credentials.

^d^Soldiers above the rank of E4 are not promoted on a set schedule.

^e^Combat Arms includes Combat Arms and Special Forces soldiers.

**p* < .05

**TABLE S6. Multivariate associations of documented psychiatric diagnoses^a^ (day of and prior to major depression diagnosis) of active-duty Regular U.S. Army enlisted soldiers with documented suicide attempt within 30 days following initial depression diagnosis^b^**

|  | **OR^c^** | | **(95% CI^c^)** |
| --- | --- | --- | --- |
| **Psychiatric Diagnoses** |  | |  |
| **ADHD/ Learning Disorders** |  | |  |
| Day of suicidal ideation |  | |  |
| No | 1.0 | | – |
| Yes | 1.1 | | (0.6–2.0) |
| χ²_1_ | 0.06 | | |
| Prior to suicidal ideation |  | |  |
| No | 1.0 | | – |
| Yes | 0.9 | | (0.6–1.5) |
| χ²_1_ | 0.10 | | |
| **Adjustment Disorder** |  | |  |
| Day of suicidal ideation |  | |  |
| No | 1.0 | | – |
| Yes | 1.6* | | (1.1–2.4) |
| χ²_1_ | 5.39* | | |
| Prior to suicidal ideation |  | |  |
| No | 1.0 | | – |
| Yes | 1.2 | | (1.0–1.5) |
| χ²_1_ | 2.64 | | |
| **Alcohol Use Disorder** |  | |  |
| Day of suicidal ideation |  | |  |
| No | 1.0 | | – |
| Yes | 1.7* | | (1.2–2.4) |
| χ²_1_ | 10.36* | | |
| Prior to suicidal ideation |  | | |
| No | 1.0 | – | |
| Yes | 1.5* | (1.2–2.0) | |
| χ²_1_ | 9.85* | | |
| **Anxiety Disorder** |  | |  |
| Day of suicidal ideation |  | |  |
| No | 1.0 | | – |
| Yes | 1.4* | | (1.1–1.7) |
| χ²_1_ | 6.50* | | |
| Prior to suicidal ideation |  | |  |
| No | 1.0 | | – |
| Yes | 1.0 | | (0.8–1.2) |
| χ²_1_ | 0.00 | | |
| **Bipolar Disorder** |  | |  |
| Day of suicidal ideation |  | |  |
| No | 1.0 | | – |
| Yes | 5.3* | | (2.7–10.4) |
| χ²_1_ | 23.43* | | |
| Prior to suicidal ideation |  | |  |
| No | 1.0 | | – |
| Yes | 1.1 | | (0.4–3.0) |
| χ²_1_ | 0.05 | | |
| **Conduct Disorder/ Oppositional Defiant Disorder** |  | |  |
| Day of suicidal ideation |  | |  |
| No | 1.0 | | – |
| Yes | 2.5 | | (0.6–10.3) |
| χ²_1_ | 1.67 | | |
| Prior to suicidal ideation |  | |  |
| No | 1.0 | | – |
| Yes | 1.2 | | (0.3–4.9) |
| χ²_1_ | 0.07 | | |
| **Dysthymic Disorder/**  **Neurasthenia/Depression NOS** |  | |  |
| Day of suicidal ideation |  | |  |
| No | 1.0 | | – |
| Yes | 1.8* | | (1.3–2.5) |
| χ²_1_ | 11.90* | | |
| Prior to suicidal ideation |  | |  |
| No | 1.0 | | – |
| Yes | 1.2 | | (1.0–1.5) |
| χ²_1_ | 2.79 | | |
| **Eating Disorders** |  | |  |
| Day of suicidal ideation |  | |  |
| No | 1.0 | | – |
| Yes | – | | – |
| χ²_1_ | 0.002 | | |
| Prior to suicidal ideation |  | |  |
| No | 1.0 | | – |
| Yes | 1.0 | | (0.1–7.1) |
| χ²_1_ | 0.00 | | |
| **Stressors/Adversities & Marital Problems** |  | |  |
| Day of suicidal ideation |  | |  |
| No | 1.0 | | – |
| Yes | 1.3* | | (1.0–1.8) |
| χ²_1_ | 4.36* | | |
| Prior to suicidal ideation |  | |  |
| No | 1.0 | | – |
| Yes | 1.0 | | (0.8–1.2) |
| χ²_1_ | 0.01 | | |
| **Non-Affective Psychosis** |  | |  |
| Day of suicidal ideation |  | |  |
| No | 1.0 | | – |
| Yes | 2.6* | | (1.3–5.0) |
| χ²_1_ | 7.60* | | |
| Prior to suicidal ideation |  | |  |
| No | 1.0 | | – |
| Yes | 0.2 | | (0.0–1.7) |
| χ²_1_ | 2.10 | | |
| **Organic Mental Disorders** |  | |  |
| Day of suicidal ideation |  | |  |
| No | 1.0 | | – |
| Yes | 1.9 | | (0.7–5.1) |
| χ²_1_ | 1.54 | | |
| Prior to suicidal ideation |  | |  |
| No | 1.0 | | – |
| Yes | 1.0 | | (0.5–1.9) |
| χ²_1_ | 0.001 | | |
| **Other Disorder^d^** |  | |  |
| Day of suicidal ideation |  | |  |
| No | 1.0 | | – |
| Yes | 7.0* | | (4.9–9.9) |
| χ²_1_ | 118.47* | | |
| Prior to suicidal ideation |  | |  |
| No | 1.0 | | – |
| Yes | 1.3 | | (0.8–2.2) |
| χ²_1_ | 1.06 | | |
| **Other Impulse Control Disorders** |  | |  |
| Day of suicidal ideation |  | |  |
| No | 1.0 | | – |
| Yes | 1.5 | | (0.5–4.8) |
| χ²_1_ | 0.54 | | |
| Prior to suicidal ideation |  | |  |
| No | 1.0 | | – |
| Yes | 0.7 | | (0.2–2.3) |
| χ²_1_ | 0.28 | | |
| **Personality Disorders** |  | |  |
| Day of suicidal ideation |  | |  |
| No | 1.0 | | – |
| Yes | 3.2* | | (1.7–6.3) |
| χ²_1_ | 11.78* | | |
| Prior to suicidal ideation |  | |  |
| No | 1.0 | | – |
| Yes | 1.9 | | (1.0–3.6) |
| χ²_1_ | 3.41 | | |
| **Sexual Disorders** |  | |  |
| Day of suicidal ideation |  | |  |
| No | 1.0 | | – |
| Yes | 1.4 | | (0.2–10.4) |
| χ²_1_ | 0.13 | | |
| Prior to suicidal ideation |  | |  |
| No | 1.0 | | – |
| Yes | 0.6 | | (0.2–1.5) |
| χ²_1_ | 1.30 | | |
| **Sleep Disorders** |  | |  |
| Day of suicidal ideation |  | |  |
| No | 1.0 | | – |
| Yes | 0.4* | | (0.1–1.0) |
| χ²_1_ | 3.95* | | |
| Prior to suicidal ideation |  | |  |
| No | 1.0 | | – |
| Yes | 1.2 | | (0.9–1.6) |
| χ²_1_ | 1.00 | | |
| **Somatoform/Dissociative Disorders** |  | |  |
| Day of suicidal ideation |  | |  |
| No | 1.0 | | – |
| Yes | 0.6 | | (0.1–4.4) |
| χ²_1_ | 0.24 | | |
| Prior to suicidal ideation |  | |  |
| No | 1.0 | | – |
| Yes | 1.7* | | (1.0–2.8) |
| χ²_1_ | 4.60* | | |
| **TBI/Postconcussion Syndrome** |  | |  |
| Day of suicidal ideation |  | |  |
| No | 1.0 | | – |
| Yes | – | | – |
| χ²_1_ | 0.002 | | |
| Prior to suicidal ideation |  | |  |
| No | 1.0 | | – |
| Yes | 1.6 | | (1.0–2.7) |
| χ²_1_ | 3.21 | | |
| **Tobacco Use Disorder** |  | |  |
| Day of suicidal ideation |  | |  |
| No | 1.0 | | – |
| Yes | 1.7* | | (1.2–2.3) |
| χ²_1_ | 9.18* | | |
| Prior to suicidal ideation |  | |  |
| No | 1.0 | | – |
| Yes | 1.1 | | (0.9–1.4) |
| χ²_1_ | 1.42 | | |
| **Traumatic Stress** |  | |  |
| Day of suicidal ideation |  | |  |
| No | 1.0 | | – |
| Yes | 2.9* | | (1.6–5.2) |
| χ²_1_ | 11.46* | | |
| Prior to suicidal ideation |  | |  |
| No | 1.0 | | – |
| Yes | 1.1 | | (0.7–1.7) |
| χ²_1_ | 0.24 | | |
| **PTSD** |  | |  |
| Day of suicidal ideation |  | |  |
| No | 1.0 | | – |
| Yes | 1.2 | | (0.8–1.6) |
| χ²_1_ | 0.76 | | |
| Prior to suicidal ideation |  | |  |
| No | 1.0 | | – |
| Yes | 1.2 | | (0.9–1.7) |
| χ²_1_ | 1.52 | | |
| **Drug-Induced Mental Disorders** |  | |  |
| Day of suicidal ideation |  | |  |
| No | 1.0 | | – |
| Yes | 2.0 | | (0.8–4.9) |
| χ²_1_ | 2.30 | | |
| Prior to suicidal ideation |  | |  |
| No | 1.0 | | – |
| Yes | 1.7* | | (1.0–2.9) |
| χ²_1_ | 4.39* | | |
| **Non-Dependent Drug Abuse** |  | |  |
| Day of suicidal ideation |  | |  |
| No | 1.0 | | – |
| Yes | 1.6 | | (0.9–3.0) |
| χ²_1_ | 2.50 | | |
| Prior to suicidal ideation |  | |  |
| No | 1.0 | | – |
| Yes | 1.3 | | (0.8–2.1) |
| χ²_1_ | 1.26 | | |
| **Drug Dependence** |  | |  |
| Day of suicidal ideation |  | |  |
| No | 1.0 | | – |
| Yes | 0.5 | | (0.2–1.5) |
| χ²_1_ | 1.52 | | |
| Prior to suicidal ideation |  | |  |
| No | 1.0 | | – |
| Yes | 1.0 | | (0.5–1.9) |
| χ²_1_ | 0.001 | | |
| **Indicator of Impulsivity & Risky Behavior** |  | |  |
| Day of suicidal ideation |  | |  |
| No | 1.0 | | – |
| Yes | – | | – |
| χ²_1_ | 0.001 | | |
| Prior to suicidal ideation |  | |  |
| No | 1.0 | | – |
| Yes | 0.6 | | (0.2–1.8) |
| χ²_1_ | 0.85 | | |

^a^Table S3 includes all ICD-9-CM and ICD-10 codes associated with each mental disorder

^b^A series of multivariate models that examined each mental disorder separately, adjusting for socio-demographics (gender, current age, race, education, and marital status) and service-related characteristics, were conducted.

^c^OR = Odds ratio; CI = Confidence interval

^d^Among soldiers who were diagnosed with a disorder classified as “Other,” 33/37 (89.2%) were identified by the ICD-9-CM code 300.9 (Unspecified nonpsychotic mental disorder)

**p* < .05
